# A stratified human colon model reveals SCFA cross-feeding-driven host–microbe dynamics associated with epithelial and immune responses

**DOI:** 10.1080/19490976.2026.2749954

**Published:** 2026-10-23

**Authors:** Taewon Kim, Joon-Young Park, Minae Kim, Na Young Kim, Seol-A Jang, Han-Gyeol Park, Dong-Woo Lee, Keunwook Lee, Bong-Soo Kim, Nam Joo Kang

**Affiliations:** a Department of Biotechnology, The Catholic University of Korea, Bucheon, Republic of Korea; b Department of Life Science, Hallym University, Chuncheon, Gangwon-do, Republic of Korea; c Department of Biomedical Sciences, Hallym University, Chuncheon, Gangwon-do, Republic of Korea; d Department of Biotechnology, Yonsei University, Seoul, Republic of Korea; e Department of Nutritional Science and Food Management, Ewha Womans University, Seoul, Republic of Korea; f Global Food and Nutrition Research Institute, Ewha Womans University, Seoul, Republic of Korea; g Graduate Program in System Health Science and Engineering, Ewha Womans University, Seoul, Republic of Korea

**Keywords:** Host–microbe interactions, SCFA cross-feeding, *Anaerostipes caccae*, *Bifidobacterium bifidum*, three-dimensional co-culture model, Colon-epithelial barrier function, mitochondrial bioenergetics

## Abstract

Host–microbe interactions are essential for maintaining intestinal homeostasis, yet most *in vitro* models lack physiological oxygen gradients, mucosal architecture, and reproducibility. We developed the Mucosal Mimetic 3D–Host Aerobic Microbiota Anaerobic Co-culture (Mm 3D-HaeMan) system, a colon-mimetic, oxygen-stratified platform enabling stable co-culture of colonic epithelial cells, human peripheral blood mononuclear cells (hPBMCs), and obligate anaerobes. Oxygen profiling confirmed a stable gradient consistent with human colonic stratification. Using a canonical short-chain fatty acid (SCFA) cross-feeding pair (*Anaerostipes caccae* and *Bifidobacterium bifidum, AcBb* consortium), we performed integrated, multilayered analyzes of the host-microbe interface. Co-culture with the SCFA-producing *AcBb* consortium was associated with enhanced epithelial-mitochondrial respiration, maintenance of transepithelial resistance, tight junction protein expression without barrier disruption, and rebalanced cytokine profiles. Host transcriptomics identified 41 differentially expressed genes (15 upregulated, 26 downregulated) associated with homeostatic-relevant epithelial responses. Simultaneously, bacterial metatranscriptomics revealed 29 differentially expressed gene families; notably, *glaK* was enriched in bacteria-only cultures; *SORD*, *nuoG*, and *nuoE* were specifically induced during co-culture, indicating host-associated bacterial transcriptional changes. The compartmentalized design permits independent sampling for layer-resolved multi-omics and functional readouts. These findings establish Mm 3D-HaeMan as a physiologically grounded platform for dissecting bidirectional host–microbe responses and immune-associated interactions. This model offers broad applicability for mechanistic research and translational screening of probiotics and microbiome-based therapeutics.

## Graphical abstract

**Figure:**
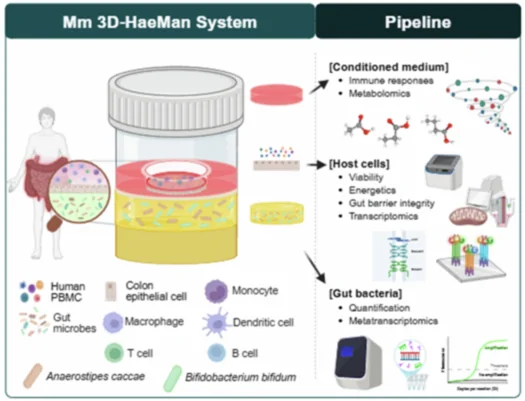

## Introduction

The human microbiome plays a fundamental role in health and disease through dynamic interactions with the host. Accumulating evidence demonstrates that these interactions are essential for the development of the immune system,^1^ ^,^ ^2^ physiological processes such as aging,^3^ pathogenesis of diverse diseases,^4-6^ and efficacy of therapeutic interventions.^7^ Among host-microbiome interfaces, the intestine is the primary site of bidirectional cross-talk between epithelial and immune cells and resident microbes.^8^ The gut microbiome constitutes a functionally complex ecosystem that produces a broad spectrum of bioactive metabolites, including vitamins, neurotransmitters, and short-chain fatty acids (SCFAs).^9^ ^,^ ^10^ Proper regulation of host-microbe interactions is therefore considered important for maintaining intestinal and systemic homeostasis. Despite their importance, the mechanistic dissection of host-microbiome interactions remains challenging in conventional experimental systems. Animal models exhibit substantial interspecies differences in intestinal physiology, microbiome, and dietary metabolism, limiting translational relevance to humans.^11^ ^,^ ^12^ Similarly, traditional static *in vitro* models and intestinal organoid systems do not fully recapitulate key dynamic features of the intestinal microenvironment, including mechanical forces, microbial complexity, and three-dimensional mucosal architecture.^13^ ^,^ ^14^ Moreover, oxygen is a primary ecological constraint in the colon that shapes the microbiome and metabolic products; a failure to reproduce mucosal oxygen stratification often prevents the stable co-culture of strict anaerobes and complicates metabolite-driven host readouts.

Although *in vitro* host-microbe models offer advantages over animal systems regarding cost and experimental control, current platforms remain constrained by technical and biological limitations. A wide range of systems have been developed, including Transwell co-cultures,^15^ Host–Microbiota Interaction (HMI^TM^) modules,^16^ Human oxygen–Bacteria anaerobic (HoxBan) systems,^17^ gut-on-a-chip devices,^18^ the Human–Microbial crosstalk (HuMiX) microfluidic platform,^19^ and microaerobic vertical diffusion chamber (VDC) model.^20^ Each of these platforms has contributed important methodological advances: oxygen-stratified systems such as HoxBan, HuMiX, and the microaerobic vertical diffusion chamber (VDC) model have demonstrated the feasibility of co-culturing strict anaerobes or microaerobic bacteria with human intestinal epithelial cells under controlled oxygen conditions, while microfluidic platforms have enabled precise environmental control and real-time physiological monitoring. However, platforms requiring microfluidic instrumentation or specialized anaerobic equipment may present practical constraints on experimental throughput, multi-omics biomass yield, and accessibility, depending on the laboratory infrastructure. Moreover, most prior platforms have used carcinoma-derived epithelial cell lines and have not incorporated circulating immune components alongside strict anaerobes. Additionally, simultaneous layer-resolved profiling of both host and microbial transcriptomes has not been reported for these systems.

To functionally evaluate whether a developed host-microbe interaction system captures key features of the gut metabolic environment, the incorporation of representative microbial interactions is informative. In the healthy colon, primary fermenters degrade complex polysaccharides into acetate and other intermediates, which are subsequently utilized by secondary fermenters to generate butyrate via metabolic cross-feeding networks.^21^ ^,^ ^22^ Among these cross-feeding interactions, the relationship between Bifidobacterium species and *Anaerostipes caccae* has been directly characterized in co-culture experiments, in which *B. bifidum*-derived lactate and acetate serve as substrates for time-dependent butyrate accumulation by *A. caccae*, providing experimental confirmation of the metabolic dependency of this consortium.^23^ Accordingly, establishing stable SCFA cross-feeding consortia and reproducing physiological acetate-propionate-butyrate profiles represent a meaningful functional criterion for evaluating the metabolic relevance of *in vitro* co-culture systems represent a meaningful functional criterion for evaluating the metabolic relevance of *in vitro* co-culture systems. Although several studies have investigated SCFA cross-feeding, most have focused on microbe-microbe interactions or applied single SCFA producers or culture supernatants to simplified epithelial co-culture systems.^24-26^ A platform that integrates these biological components within a standard laboratory-compatible format, enabling simultaneous multi-omics output from both host and microbial compartments, has not yet been described.

Here, we present Mm 3D-HaeMan (Mucosal Mimetic 3D–Host Aerobic Microbiota Anaerobic) co-culture platform, which builds on the established principle of oxygen-stratified host–microbe co-culture^17^ ^,^ ^27^ and extends it through three combinatorial advances not previously implemented in a single experimental system. 1) Mm 3D-HaeMan supports stable strict anaerobic co-culture with sufficient multi-omics biomass recovery using standard cell-culture infrastructure, without requiring microfluidic fabrication or specialized gas-delivery hardware, substantially lowering the technical barrier to entry and facilitating broader laboratory adoption compared to platforms such as HuMiX and gut-on-a-chip. 2) The system integrates a non-tumorigenic colonic epithelial cell line (CCD841CoN), human peripheral blood mononuclear cells (hPBMC) as a circulating immune surrogate, and a defined SCFA cross-feeding consortium of strict anaerobes within a single three-layer configuration. This combination has not previously been reported in oxygen-stratified co-culture platforms. 3) Mm 3D-HaeMan enables the simultaneous recovery of biomass for host transcriptomics and bacterial metatranscriptomics from the same experimental run, enabling layer-resolved multi-omics characterization of epithelial, immune, and microbial responses within a unified analytical framework, which reduces experimental complexity and broadens the accessibility for multi-omics studies.

It is important to note that the Mm 3D-HaeMan system is designed as a reductionist experimental system that prioritizes mechanistic tractability and reproducibility over full tissue complexity. CCD841CoN cells, while non-tumorigenic, lack goblet cells, absorptive differentiation, and three-dimensional tissue organization and do not fully recapitulate the cellular heterogeneity of the colonic epithelium. Similarly, hPBMCs represent a circulating immune fraction rather than a gut-resident mucosal immune population, and the cytokine responses derived from this system should be interpreted accordingly. Rather than recapitulating all features of the colonic mucosa, Mm 3D-HaeMan is intended to enable controlled interrogation of specific host–microbe interaction programs under defined biological and metabolic conditions. We present Mm 3D-HaeMan not as a conceptual departure from prior work but as a practically accessible and analytically integrated combinatorial advance within an established framework.

## Materials and methods

### Host cells

CCD841CoN cells, a normal human colon epithelial cell line, and hPBMCs were obtained from the American Type Culture Collection (ATCC; Manassas, VA, USA). Minimum essential medium (MEM) powder, MEM non-essential amino acids (NEAA) solution, sodium pyruvate, fetal bovine serum (FBS), penicillin/streptomycin (P/S), trypsin-ethylene-diamine-tetra-acetic acid (trypsin-EDTA), and 0.4% trypan blue solution were obtained from Thermo Fisher Scientific (Waltham, MA, USA). CCD841CoN cells and hPBMCs were maintained in Eagle's Minimum Essential Medium (EMEM) supplemented with 10% (v/v) FBS and 1% (v/v) P/S, at 37 °C in a humidified 5% CO_2_ atmosphere. EMEM was prepared by dissolving MEM powder, NEAA, sodium pyruvate, and sodium bicarbonate (NaHCO_3_; Duksan General Science, Seoul, South Korea) according to the manufacturer’s instructions. CCD841CoN cells were subcultured at 80–90% confluence, and experiments were performed using passages 16–24. The hPBMCs were thawed and used immediately without further passaging.

### Establishment of the Mm 3D-HaeMan co-culture system

#### Selection of culture medium for SCFA cross-feeding bacteria

Representative SCFA cross-feeding bacteria and gut-derived strains, *Anaerostipes caccae* KCTC 15019, *Bifidobacterium bifidum* YF217, *Faecalibacterium prausnitzii*, and *Bifidobacterium adolescentis*, were used in this study. To determine a suitable medium for their co-cultivation, both strains were cultured anaerobically in four growth media: de Man, Rogosa, and Sharpe (MRS; BD Biosciences, Franklin Lakes, NJ, USA), Tryptic Soy Broth (TSB; BD Biosciences), Reinforced Clostridial Medium (RCM; Oxoid, Basingstoke, UK), and modified Peptone Yeast Extract Glucose (PYG; modified DSMZ 104 medium; following DSMZ instructions). Cultures were maintained under anaerobic conditions (10% H_2_, 10% CO_2_, and 80% N_2_) using an anaerobic workstation (Don Whitley Scientific Limited, West Yorkshire, UK).

Both monoculture and co-culture conditions were evaluated. For monocultures, a 1% (v/v) aliquot of the subculture was inoculated into each medium. For co-cultures, 0.5% (v/v) of each strain was inoculated (for a total of 1%). Cultures were incubated for 24 h, and bacterial growth was monitored at 3-h intervals by measuring the optical density at 600 nm (OD₆₀₀). All experiments were performed in biological triplicate.

#### Bacterial pre-culture procedures

*A. caccae* or *B. bifidum* was streaked onto TSB-agar (Bacto™ agar; BD Biosciences) and cultivated in BD GasPak™ EZ pouch systems (BD Biosciences). A single colony of *A. caccae* or *B. bifidum* was inoculated into 10 mL of TSB and grown to the mid-exponential phase (OD_600_ ≈ 0.4–0.6). To maintain the growth rate of both bacteria, a 1-mL aliquot of the exponential-phase culture was transferred into 10 mL of fresh TSB and grown again to an OD_600_ ≈ 0.4–0.6 prior to inoculation into the Mm 3D-HaeMan system.

#### Host cell preparation and pre-culture

CCD841CoN epithelial cells were seeded onto the outer membrane of 0.4-μm pore and 6-well SPLInsert™ Standing inserts (SPL Life Sciences, Pocheon, South Korea) pre-coated with 0.1% (w/v) gelatin at a density of 4 × 10^4^ cells/cm^2^. On the following day, hPBMCs were seeded into 24-well plates (SPL Life Sciences) at 1 × 10^6^ cells/well. After 24 h, the inserts were inverted, and 2 mL of fresh complete EMEM was added to both the apical side (inside the inserts) and the basolateral compartment of a 6-well plate. One hour prior to co-culture, the CCD841CoN culture medium was replaced with antibiotic-free EMEM supplemented with 10% FBS.

#### Host-microbe co-culture in the Mm 3D-HaeMan system

Inside an anaerobic chamber, 40 mL of anaerobic TSB containing 0.5% (w/v) Gelrite (Sigma-Aldrich, Burlington, MA, USA) was dispensed into aseptic bottles and allowed to solidify to form the base layer. Exponential-phase *A. caccae* and *B. bifidum* were mixed and inoculated into 40 mL of anaerobic TSB containing 0.2% (w/v) Gelrite (cooled to approximately 50–52°C), which was then overlaid onto the solidified base. After the bacterial layer had solidified, a 2.5 mL mucus layer composed of 0.1% (w/v) mucin type III (Sigma-Aldrich), 0.3% (w/v) Gelrite, and 0.03% (w/v) magnesium chloride (Sigma-Aldrich) was applied. The mucus layer was further deoxygenated by flushing with an anaerobic gas mixture (10% H_2_, 10% CO_2_, and 80% N_2_) and supplemented with 0.004% (w/v) Na_2_S (Sigma-Aldrich). Once the mucus layer had solidified, antibiotic-free EMEM (10% FBS) was gently added on top to facilitate host-microbe co-culture. Pre-cultured CCD841CoN cells and hPBMCs were transferred into the aseptic bottles (New Kukje Scientific Corporation, Namyangju, South Korea) and incubated for 18 h at 37 °C in a humidified 5% CO_2_ atmosphere.

### Quantification of SCFAs by ultra-high performance liquid chromatography-Tandem mass spectrometry analysis

To assess the SCFA production resulting from cross-feeding between *A. caccae* and *B. bifidum* under different culture conditions, the supernatants were collected after centrifugation at 10,000 × *g* for 10 min. For metabolite extraction, 100 μL of supernatant was mixed with internal standards and 400 μL of acetonitrile, vortexed for 5 min, and centrifuged at 10,000 × *g* for 5 min. The clarified supernatant was filtered through a 0.22-μm polytetrafluoroethylene syringe filter prior to ultra-high performance liquid chromatography-tandem mass spectrometry (UHPLC-MS/MS) analysis.

SCFA standards were prepared from 100 mM stock solutions diluted in acidified Milli-Q® water (Millipore, Burlington, MA, USA). Stock solutions were stored at −20 °C, and fresh standard curves were generated daily by serial dilution (10 mM–0.001 mM) immediately prior to analysis.

SCFAs were analyzed using a QTRAP 5500 mass spectrometer (Applied Biosystems, Foster City, CA, USA) coupled to an ExionLC™ Series UHPLC system (Shimadzu, Tokyo, Japan) equipped with an electrospray ionization source operating in positive and negative ion modes. Chromatographic separation was achieved using a Waters ACQUITY UPLC HSS T3 C18 column (2.1 × 50 mm, 1.8 μm) maintained at 40 °C, with the autosampler temperature held at 4 °C. A 5-μL injection volume was used. Mobile phases consisted of 0.1% (v/v) formic acid in water (A) and 0.1% (v/v) formic acid in acetonitrile (B). A gradient elution from 0% to 60% B over 4 min was applied, with a total run time of 10 min, at a flow rate of 0.5 mL/min. Nitrogen was used as the nebulizer, desolvation, and collision gas.

SCFAs were quantified in multiple reaction monitoring (MRM) mode. Precursor-product ion pairs and collision energies were optimized using Analyst 1.7.1 software. The ion source was operated with the following parameters: temperature, 600 °C; curtain gas, 30 psi; collision gas, medium; ion spray voltage, +5500 V (positive mode) or −4500 V (negative mode); GS1, 60 psi; and GS2, 50 psi. SCFAs were identified and quantified based on retention times and MRM transitions relative to authentic and internal standards. Final concentrations were reported in millimolar (mM) units.

### Quantification of gut bacteria by quantitative real-time polymerase chain reaction

For culture media selection experiments, *A. caccae*, *B. bifidum*, *F. prausnitzii*, and *B. adolescentis* were anaerobically cultured in four culture media at 37 °C for 24 h, and the bacterial load was quantified. Following incorporation into the Mm 3D-HaeMan system, the bacterial load was measured after 18 h of co-culture to match the system’s optimized incubation period. After cultivation, cultures were centrifuged at 10,000 rpm for 10 min, and the resulting pellets were used for genomic DNA extraction with the RNeasy PowerMicrobiome Kit (Qiagen, Hilden, Germany) according to the manufacturer’s instructions. For samples obtained from the Mm 3D-HaeMan system, total DNA and RNA were extracted from 0.25 g of the semi-solid bacterial layer using the same extraction kit. Prior to extraction, samples were thoroughly homogenized, and 0.25 g of the homogenate was accurately weighed for downstream processing.

Quantification of *A. caccae*,^28^ *B. bifidum,* ^29^ *F. prausnitzii,* ^30^ and *B. adolescentis* ^29^ was performed using species-specific quantitative real-time polymerase chain reaction (qPCR) with validated primers. Amplifications were conducted using a Thermal Cycler Dice Real-Time System III (Takara Bio, Shiga, Japan) in 25 μL volumes containing 12.5 μL of 2 × TB Green Premix Ex Taq (Tli RNaseH Plus; Takara Bio), 2 μM of each primer, and 2 μL of DNA template (10-fold dilution series of sample DNA) or distilled water (negative control). The amplification conditions consisted of initial denaturation at 95 °C for 30 s, followed by 35 cycles of denaturation at 95 °C for 5 s and annealing at 60 °C for 30 s. Bacterial abundance was calculated by comparing cycle threshold (Ct) values to standard curves generated from parallel reactions of 10-fold serial dilutions (1 × 10^1^–1 × 10^7^ CFU/mL) of genomic DNA from known colony-forming units (CFUs) of each species. Standard curves showed high linearity (R^2^ ≥ 0.99). Bacterial growth rates (*μ*) were calculated during the exponential phase using OD₆₀₀ measurements and qPCR-estimated CFU values, applying the following equation:

$$\mu =(\ln(N_{t}/N_{0}))/t$$

where *N*_*t*_ and *N*_*0*_ represent bacterial abundance at the end and beginning of the time interval (*t*), respectively.

### Measurement of oxygen profiles in the Mm 3D-HaeMan system

Dissolved oxygen concentrations within the liquid, mucus, and semi-solid layers of the Mm 3D-HaeMan system were measured using a Microx 4 oxygen sensor equipped with a needle-type oxygen PSt7 probe (PreSens, Bavaria, Germany), following the manufacturer's instructions. Measurements were conducted at 0 and 18 h during co-culture. The probe was lowered vertically in 1-mm increments from the top of the epithelial cell culture medium to the bottom of the semi-solid layer. All measurements were performed in triplicate.

### Crystal violet staining and quantification of epithelial cell density

CCD841CoN cells cultured on inserts were washed with phosphate-buffered saline (PBS) and fixed in 4% (w/v) paraformaldehyde (BioSesang, Yongin, South Korea) for 10 min at room temperature (RT). After two additional washes with PBS, cells were stained with 1% (w/v) crystal violet (Sigma-Aldrich) for 15 min at RT. Excess dye was removed by PBS washes, and the inserts were air-dried. Stained cells were imaged using a light microscope (Motic, Kowloon, Hong Kong) equipped with a Moticam digital camera, and images were processed using Motic Image Plus v.3.0.

For quantification, bound dye was eluted with 95% (v/v) ethanol for 30 min at RT, and absorbance was measured at 570 nm using a microplate reader (Sunrise; Tecan, Männedorf, Switzerland). Cell density was calculated from a calibration curve generated from standard samples (serially diluted from 2.5 × 10^4^ to 0.156 × 10^4^ cell/cm^2^) prepared under conventional culture (CC) conditions, in which CCD841CoN cells were maintained in EMEM under standard aerobic conditions in 6-well inserts without Mm 3D-HaeMan configuration.

### Trypan blue viability assay

After 24 h of co-culture, CCD841CoN cells were washed with warm (37 °C) PBS and incubated with 0.25% (w/v) trypsin-EDTA at 37 °C for 5 min. Cells were collected in complete EMEM (10% FBS, 1% P/S), pelleted by centrifugation at 8,000 rpm for 3 min at RT, and resuspended in fresh medium. Suspended cells were mixed 1:1 with 0.4% (w/v) trypan blue solution; viable (unstained) and non-viable (blue-stained) cells were counted using a Countess II FL Automated Cell Counter (Thermo Fisher Scientific). Viability was expressed as the percentage of unstained cells.

### MTT-based assessment of cellular metabolic activity

To assess whether the observed changes in cellular metabolic activity required viable bacterial components or could be attributed to heat-killed bacteria or soluble metabolites, CCD841CoN cells were seeded at 1 × 10^4^ cells/well in 96-well plates and cultured overnight at 37 °C in a humidified incubator with 5% CO₂.

Bacterial preparations were generated from *A. caccae*, *B. bifidum*, and the *AcBb* consortium, each inoculated at a density equivalent to that of the *AcBb*-L condition (*A. caccae*: 250 cells; *B. bifidum*: 2,500 cells in total) into 40 mL of anaerobic TSB broth and cultured anaerobically at 37 °C for 18 h. For live bacterial preparations, culture turbidity was measured by the OD_600_ after cultivation, and bacterial concentrations were determined. Bacterial suspensions were mixed with an equal volume of 50% (v/v) glycerol solution to a final glycerol concentration of 25% and stored at −80 °C. Prior to host cell treatment, cryopreserved live bacteria were thawed and centrifuged at 12,000 rpm for 2 min at room temperature. The cryopreservation medium was removed, and the bacterial pellet was washed once with PBS and resuspended in serum-free host cell culture medium at the following final treatment concentrations: *A. caccae*, 9.5 × 10^6^ cells/well; *B. bifidum*, 8.2 × 10^5^ cells/well; *AcBb* consortium, 1.3 × 10^7^ cells/well.

Heat-killed bacterial preparations were prepared from aliquots of the same live bacterial suspension prepared at equivalent concentrations. Aliquots were transferred to microcentrifuge tubes and heated at 90 °C for 30 min in a heating block, after which glycerol was added to a final concentration of 25% and the samples were stored at −80 °C. Host cell treatment of the heat-killed preparations followed the same procedure as that used for live bacteria.

Culture supernatants were prepared by centrifuging 18 h bacterial cultures at 3,000 × *g* for 10 min at room temperature, followed by filtration through 0.2-μm membrane filters (Millipore) to remove residual bacteria, and stored at −80 °C until use. For host cell treatment, the supernatants were diluted 1:1 (v/v) with serum-free host cell culture medium to match the volumetric ratio applied to the bacterial cell preparations. TSB broth diluted 1:1 (v/v) in serum-free medium served as the blank (medium).

All preparations were applied to CCD841CoN cells, which were incubated for 18 h at 37 °C with 5% CO₂. Following incubation, 20 μL of 3-(4,5-dimethylthiazol-2-yl)-2,5-diphenyltetrazolium bromide (MTT) solution (final concentration: 1 mg/mL; Sigma-Aldrich) was added to each well and incubated for 2 h. Formazan crystals were dissolved by addition of dimethyl sulfoxide, and absorbance was measured at 570 nm using a microplate reader (Sunrise, Tecan). Cellular metabolic activity was expressed as relative MTT activity normalized to the absorbance of untreated control cells and presented as a percentage of the control.

### Measurement of transepithelial electrical resistance and voltage

Epithelial barrier integrity was assessed by measuring transepithelial electrical resistance (TEER) and transepithelial voltage (V*te*) using a Millicell ERS-3.0 Digital Voltohmmeter (Millipore). Measurements were performed on CCD841CoN cells seeded on 6-well SPLInsert (SPL Life Sciences) at two time points: immediately prior to co-culture initiation (0 h) and at the end of the 18 h co-culture period (18 h). A blank control comprising inserts containing culture medium only without cells was included in each independent experiment for baseline correction. TEER and voltage were calculated using the following formulas:

$$\mathrm{TEER}(\Omega \;{\mathrm{cm}}^{2})=(R_{\mathrm{sample}}-R_{\mathrm{blank}})\times A\mathrm{rea}$$

$$V\mathrm{oltage}(mV/{\mathrm{cm}}^{2})=(V_{\mathrm{sample}}-V_{\mathrm{blank}})/A\mathrm{rea}$$

### Immunofluorescence staining of colon epithelial markers

CCD841CoN cells cultured on 6-well insert membranes were fixed with 4% paraformaldehyde for 15 min at room temperature, washed three times with cold PBS, and permeabilized with 0.3% Triton X-100 in PBS for 10 min at room temperature. After three washes with PBS, the cells were blocked with 5% normal goat serum and 0.3% Triton X-100 in PBS for 1 h at room temperature.

Primary antibody incubation was performed sequentially. *β*-catenin primary antibody (Cell Signaling Technology, Danvers, MA, USA) was diluted 1:200 in antibody diluent solution (1% BSA and 0.3% Triton X-100 in PBS) and applied overnight at 4 °C. After three washes with PBS, Goat anti-rabbit IgG FITC secondary antibody (Invitrogen, Waltham, MA, USA) was applied at a 1:1000 dilution for 1 h at room temperature in the dark. After three PBS washes, Alexa Fluor 647-conjugated anti-ZO-1 antibody (Invitrogen) was applied at a 1:400 dilution for 2 h at room temperature in the dark. Nuclei were counterstained with DAPI (1 μg/mL in PBS) for 5 min, followed by three PBS washes.

The insert membranes were excised and mounted on glass slides using the DAKO fluorescent mounting medium (Agilent Technologies, Santa Clara, CA, USA). Images were acquired using a confocal laser scanning microscope (LSM 900; Carl Zeiss, Jena, Germany) with sequential channel detection in the following order to minimize photobleaching: AF647 (640 nm excitation, 650–750 nm detection), FITC (488 nm excitation, 495–519 nm detection), and DAPI (405 nm excitation, 410–480 nm detection). Z-stack images were acquired at 0.3–0.5 μm intervals over a range encompassing the full cell layer with 5 μm margins above and below, using a 40 × objective lens. Representative xy-plane and xz-axis orthogonal projections were generated for analysis.

### Assessment of mitochondrial function using Seahorse XFp Cell Mito Stress Test

Mitochondrial respiration in CCD841CoN cells was assessed using the Seahorse XFp Cell Mito Stress Test Kit (Agilent Technologies). CCD841CoN cells harvested from the Mm 3D-HaeMan system were resuspended in fresh medium and seeded at 3 × 10^4^ cells/well in XFp miniplates. After 24-h incubation at 37 °C with 5% CO_2_, culture medium was replaced with Seahorse XF DMEM (pH 7.4; Agilent Technologies), supplemented with 2 mM glutamine, 10 mM glucose, and 1 mM pyruvate (Agilent Technologies). Plates were equilibrated in a non-CO_2_ incubator for 1 h.

Oligomycin (port A, 1.5 μM), carbonyl cyanide-4-(trifluoromethoxy)phenylhydrazone (FCCP; port B, 1 μM), and rotenone/antimycin A (port C, 0.5 μM) were loaded into the injection port of the Seahorse XFp sensor cartridge. Oxygen consumption rate (OCR) and extracellular acidification rate (ECAR) were measured on a Seahorse XFp analyzer (Agilent Technologies) using four 4-min measurement cycles before and after each injection. After the assay, cells were stained with trypan blue and counted using a Countess II cell counter (Thermo Fisher Scientific); OCR and ECAR values were normalized to 1 × 10^4^ viable cells.

### Western blot analysis of epithelial protein expression

After 18 h of co-culture, CCD841CoN cells were washed with cold PBS and lysed in Cell Lysis Buffer (Cell Signaling Technology) supplemented with 1 mM phenylmethylsulfonyl fluoride (Sigma-Aldrich) and a protease-inhibitor cocktail (Sigma-Aldrich). Lysis was performed through three rapid freeze–thaw cycles. Each cycle consisted of immersion in liquid nitrogen followed by thawing in a 37 °C water bath and vortexing. Protein concentrations were determined using the detergent-compatible (DC) protein assay kit (Bio-Rad Laboratories, Hercules, CA, USA).

Equal amounts of protein (20–35 μg) were separated by sodium-dodecyl-sulfate-polyacrylamide-gel electrophoresis and transferred to a polyvinylidene fluoride membrane (Millipore). Membranes were blocked in 5% skim milk (KisanBio, Seoul, South Korea) for 2 h and incubated overnight at 4 °C with primary antibodies of junctional-adhesion-molecule-A (JAM-A; Invitrogen), claudin-1 (Invitrogen), and *β*-actin (Sigma-Aldrich), followed by horseradish peroxidase-conjugated secondary antibodies (GeneTex, Irvine, CA, USA) for 2 h at RT. Signals were detected using chemiluminescence reagents (Cytiva, Marlborough, MA, USA) and imaged with a ChemiDoc system (Bio-Rad Laboratories).

### Human cytokine array analysis and cytometric bead array analyzes

Conditioned media collected from host cells cultured with or without bacteria in the Mm 3D-HaeMan system were centrifuged at 500 × *g* for 10 min at 4 °C to remove debris.

For multiplex cytokine array profiling, 500 μL of clarified supernatant per sample was applied to the Proteome Profiler™ Human Cytokine Array membrane (R&D Systems, Minneapolis, MN, USA) according to the manufacturer's protocol. Signal intensity for each cytokine spot was quantified using HLImage^++^ software (Western Vision Software, Salt Lake City, UT, USA).

For the human inflammatory cytokine cytometric bead array (CBA), culture supernatants were incubated with cytokine capture beads and PE-conjugated detection antibodies, and fluorescence signals were acquired using a BD FACSCanto II flow cytometer (BD Biosciences). Cytokine concentrations were calculated from standard curves generated with recombinant cytokine standards using the FCAP Array software (v3.0; BD Biosciences). The flow cytometry data were processed using FlowJo v10 (BD Biosciences).

### Transcriptome analysis for the host cell

#### RNA sequencing

Total RNA from CCD841CoN cells collected after co-culture with bacteria in the Mm 3D-HaeMan system was extracted using the RNeasy Plus Mini Kit (Qiagen) following the manufacturer's protocol. cDNA libraries were prepared using the SMARTer Stranded RNA-Seq Kit (Takara Bio). Paired-end sequencing (2 × 101 bp) was performed on an Illumina platform by Macrogen (Seoul, South Korea).

#### Processing of transcriptomic data

Adapter trimming and quality filtering of raw reads were performed using Trimmomatic v0.39,^31^ and reads were merged using PEAR v0.9.11.^32^ Ribosomal RNA sequences were removed using SortMeRNA v4.3.7.^33^ To identify human-coding genes, the reads were aligned to the human reference genome (GRCh38) using HISAT2 v2.2.1, and gene prediction was performed using BRAKER3 v3.0.8.^34^ ^,^ ^35^ Functional annotation was obtained using eggNOG-mapper v.2.1.12 against the Uniref90 database.^36^, and Uniref90 IDs were converted to Kyoto Encyclopedia of Genes and Genomes (KEGG) Orthologys (KOs).^37^ Transcript abundance was quantified as transcripts per kilobase million (TPM) using Salmon v0.13.1.^38^ Normalization across samples was performed using the cumulative sum scaling (CSS) method.^39^

#### Differential expression and functional enrichment analysis

Differential expressions were assessed using CSS-normalized TPM values. Genes with |log_2_ fold change| ≥ 1 and an adjusted *p*-value < 0.05 in the likelihood ratio test were considered differentially expressed genes (DEGs). Statistical significance for between-group comparisons was evaluated using the Wilcoxon rank-sum test with Benjamini–Hochberg (BH) correction.

Global transcriptomic variation was visualized by principal-component-analysis (PCA) using ggplot2 in R v.4.4.3. Ninety-five percent confidence ellipses were overlaid using the *stat_ellipse* function. Volcano plots were generated using ggplot2, with DEGs color-coded (upregulated: orange; downregulated: blue). Hierarchical clustering of DEGs was performed and visualized in a heatmap using the *pheatmap* package. Expression levels of selected DEGs (CSS-normalized TPM) were visualized with box plots. Functional enrichment analysis, including Gene Ontology (GO) and KEGG pathway analysis, was conducted using the *clusterProfiler* package.

### Metatranscriptome analysis for the bacteria

#### Metatranscriptomic library preparation

Extracted total RNA extracted from the semi-solid bacterial layer of the Mm 3D-HaeMan systems was used for metatranscriptomic analysis. Ribosomal RNA was depleted using the MICROBExpress Bacterial mRNA Enrichment kit (Thermo Fisher Scientific) following the manufacturer’s instructions. Metatranscriptomic libraries were prepared using the Swift RNA Library kit (Swift Biosciences, Ann Arbor, MI, USA). After adapter ligation, purification and size selection were performed using HiAccuBead (AccuGene, San Diego, CA, USA). Indexing PCR was performed using the Swift 2S Turbo Combinatorial Dual Indexing Primer Kit (Swift Biosciences), followed by an additional purification and size selection using HiAccuBead. Library concentrations were quantified using the Qubit™ dsDNA HS Assay Kit (Thermo Fisher Scientific). Equimolar concentrations of each library sample were pooled to a final concentration of 4 nM and sequenced using Illumina MiSeq (2 × 300 bp) and NovaSeq 6000 system (2 × 250 bp) platforms.

#### Processing of metatranscriptomic sequence

Raw reads were processed using Trimmomatic v.0.39^31^ for adapter trimming and quality filtering, followed by merging with PEAR v.0.9.11.^32^ Ribosomal RNA sequences were removed using SortMeRNA v.4.3.7.^33^ To remove host-derived sequences, reads were aligned to the human reference genome (GRCh38) using BBMap v.39.19,^40^ and the mapped reads were excluded from downstream analyzes.

De novo assembly of filtered host-depleted reads was performed using MEGAHIT v.1.1.3.^41^ Coding DNA sequences (CDS) from assembled contigs were predicted using Prokka v.1.14.6.^42^ Predicted genes were clustered at 90% nucleotide identity using CD-HIT-EST to construct a non-redundant gene catalog.^43^ For species-level assignment, non-redundant genes were aligned to the species-level reference genomes of *A. caccae* and *B. bifidum* using BLASTn v.2.16.0+ with a stringent *E*-value threshold of <1e-50.^44^ The reference genomes comprised *A. caccae* NBRC 114412 and *B. bifidum* JCM 1255, which are the type strains of *A. caccae* and B. *bifidum*, respectively, retrieved from the NCBI RefSeq database. Species assignment was based on the best BLASTn hits. Genes that could not be uniquely assigned to either species were classified as shared rather than attributed to a single species.

Functional annotation was performed using eggNOG-mapper v.2.1.12 against the Uniref90 database,^36^ and Uniref90 IDs were converted into KEGG Orthologs (KOs).^37^ Transcript abundance was quantified as TPM using Salmon v.0.13.1,^38^ by mapping filtered host-depleted reads back to the non-redundant gene catalog. TPM values were normalized across samples using the CSS method.^39^

### Statistical analysis

Statistical analyzes of host cell characteristic changes were performed using Welch’s *t*-test for pairwise comparisons. For comparison involving more than two groups, one-way analysis of variance (ANOVA) was applied, whereas experiments containing two independent variables were analyzed using two-way ANOVA. Transcriptomic data were evaluated using the Wilcoxon rank-sum test, and *p*-values were adjusted using the BH method to control the false discovery rate. Unless otherwise specified, all statistical analyzes were two-tailed, and statistical significance was defined as *p* < 0.05.

Microbiome-related statistical analyzes were conducted in R v.4.4.3. Group-wise differences were assessed using multivariate association with linear models (MaAsLin2).^45^ Calculated coefficients, *p*-value, and adjusted *p*-value (*P*_*adj*_) were used for downstream interpretation. For analyzes involving more than two groups, Dunn’s *post hoc* test was applied, and *p*-values were corrected for multiple comparisons using the BH method.^46^ ^,^ ^47^

### Software

Graphical figures were created and organized using GraphPad Prism v8.0 (GraphPad Software, San Diego, CA, USA) and BioRender (BioRender.com, Toronto, ON, Canada). Statistical analyzes—including data normalization, descriptive statistics, and plotting—were performed in R v4.4.3 (R Foundation for Statistical Computing, Vienna, Austria) and Microsoft Excel 365 (Microsoft Corporation, Redmond, WA, USA). Protein band intensities were quantified using Image Lab v6.1.0 (Bio-Rad Laboratories) and ImageJ v1.46r (National Institutes of Health, Bethesda, MD, USA). Mitochondrial respiration parameters were recorded and analyzed using Wave Pro software (Agilent Technologies). HLImage++ software vPCM.25.0.0.r (Western Vision Software) was used for measuring the spot intensities of the cytokine array.

## Results

### Construction of a colon-mimetic co-culture platform with physiological oxygen stratification

To recapitulate the key spatial and physiological features of the human colonic microenvironment, we developed the Mm 3D-HaeMan system, which comprises three vertically aligned, molecularly continuous compartments: (i) a liquid compartment supporting aerobic host cells, (ii) an intermediate mucus-mimetic layer permitting molecular diffusion, and (iii) a semi-solid Gelrite matrix optimized for strict anaerobes. To model a healthy intestinal barrier and immune interface, normal human colonic epithelial CCD841CoN cells and hPBMCs were seeded on opposite surfaces of a cell culture insert to represent the epithelial and immune compartments, respectively, whereas anaerobic bacteria were inoculated into the semi-solid layer to enable spatially organized host–microbe interactions.

The co-culture was assembled using a staged workflow (Figure 1a). Gut bacteria were streaked onto agar plates and incubated under anaerobic conditions using GasPak™ EZ pouch systems until colonies were formed (Day –5). CCD841CoN cells were seeded onto the outer surface of the insert membrane and cultured to confluency (Day –3), whereas hPBMCs were stabilized in 24-well plates prior to integration into the co-culture. A single bacterial colony was inoculated into a broth culture to obtain actively growing cells (Day –2). Inserts seeded with CCD841CoN cells were inverted and equilibrated in complete medium before co-culture (Day –1). hPBMCs were transferred to the inner side of the insert one hour prior to co-culture initiation, and exponentially growing bacteria were inoculated into the Gelrite-based semi-solid medium (Day 0). Host cells and bacteria were co-cultured for 18 h, after which the bacterial layer, epithelial layer, and liquid medium were harvested independently for layer-specific microbial, host, and cytokine/metabolite analyzes (Day +1).

**Figure 1.**
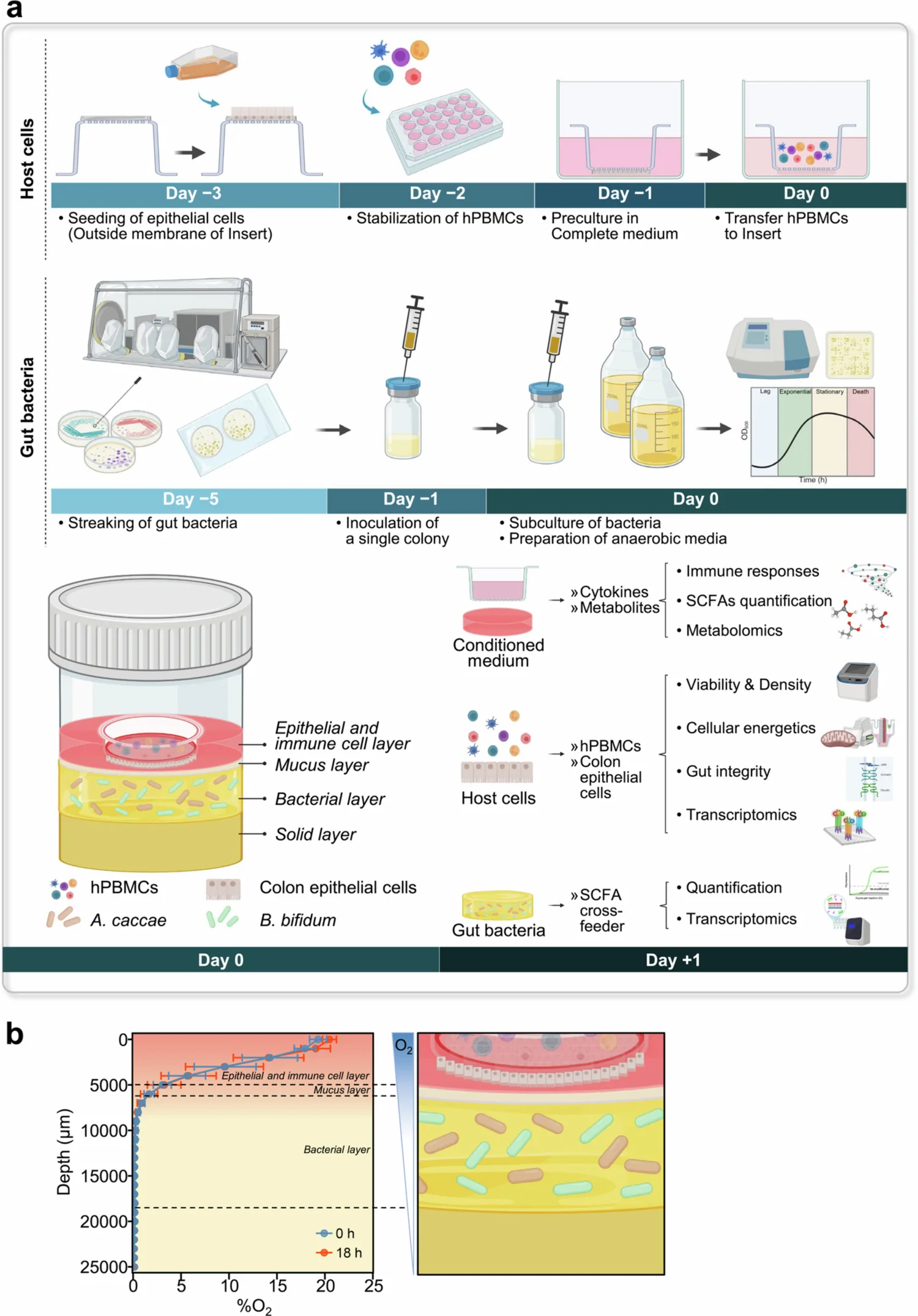
Schematic overview of the Mm 3D-HaeMan system for host–microbe co-culture. (a) Schematic representation of the Mucosal Mimetic 3D-Host Aerobic Microbiota Anaerobic Co-culture (Mm 3D-HaeMan) platform, consisting of three vertically aligned compartments: a semi-solid medium supporting anaerobic commensals (*Anaerostipes caccae* and *Bifidobacterium bifidum*), a mucus-like interface enabling host–microbe molecular exchange, and a liquid compartment containing CCD841CoN cells and human primary peripheral blood mononuclear cells (hPBMCs). Host cells are pre-cultured under aerobic conditions, whereas bacteria are anaerobically pre-grown to the exponential phase before assembly. All components are combined and co-cultured for 18 h under colon-like oxygen gradients. Each layer—microbial, epithelial, and liquid—can be independently sampled for microbial, transcriptomic, cytokine, or metabolite analyzes. (b) Oxygen profiles across layers of the Mm 3D-HaeMan system. Dissolved oxygen concentration was measured at 1,000 μm intervals from the surface of the host cell culture medium (depth = 0 μm) to the bottom of the semi-solid layer (depth = 25,000 μm) using the Microx 4 oxygen sensor at 0 and 18 h. Dashed lines indicate the boundaries between layers. Values represent mean ± SD (*n* = 4).

To validate whether the Mm 3D-HaeMan system reproduces physiological oxygen stratification, we profiled the dissolved oxygen concentrations across the vertical layers of the system. A stable oxygen gradient was consistently observed, ranging from 9.5 to 3.2% O_2_ in the epithelial/immune cell layer (3,000–5,000 μm), 3.2 to 1.7% in the mucus layer (5,000–6,000 μm), and 1.7 to 0.2% in the anaerobic bacterial layer (6,000–20,000 μm) (Figure 1b). Collectively, these results support that the Mm 3D-HaeMan system recapitulates key aspects of structural organization and oxygen stratification relevant to the human colon microenvironment, enabling simultaneous culture of aerobic host cells and strict anaerobes within a single platform.

### Optimization of co-culture conditions for SCFA cross-feeding bacteria and host cells in the Mm 3D-HaeMan system

Building on the established structural integrity and physiological oxygen gradient of the Mm 3D-HaeMan system, we next optimized the co-culture conditions to support the simultaneous growth of host cells and anaerobic bacteria. As representative human gut SCFA cross-feeders, the butyrate-producing *A. caccae* and the acetate-producing *B. bifidum* were selected. To identify an appropriate medium for co-culturing both species, bacterial growth was evaluated under monoculture and co-culture conditions across four growth media using OD_600_ measurements and species-specific qPCR analyzes (Figure S1a and b). Because OD_600_ and qPCR measure different aspects of bacterial growth, these results were interpreted as complementary rather than interchangeable measurements. OD_600_ reflects total turbidity or particle-associated biomass, whereas species-specific qPCR estimates DNA-based abundance of each bacterial species. *B. bifidum* exhibited poor growth in RCM, whereas *A. caccae* showed limited growth in PYG medium under co-culture conditions. Based on these results, MRS and TSB were selected for further evaluation.

To evaluate SCFA production by the two-strain consortium, SCFA concentrations, including acetate and butyrate, were measured in MRS and TSB media (Figure S1c). Total SCFA concentrations were significantly higher in TSB than in MRS (*p* < 0.05), supporting the selection of TSB as a more favorable medium for consortium growth and SCFA production. Species-specific qPCR further confirmed that both *A. caccae* and *B. bifidum* were maintained during co-cultivation in TSB at 12 and 24 h (Figure S1d), indicating that the subsequent metabolite profile was obtained under conditions in which both strains were present.

To further analyze metabolic interactions between the two strains, acetate and butyrate concentrations were compared among *A. caccae* monoculture, *B. bifidum* monoculture, and *AcBb* co-culture conditions in TSB (Figure S1e). At 24 h, net acetate accumulation showed a modest but non-significant decrease in the *AcBb* co-culture relative to monoculture. In contrast, butyrate accumulation was significantly greater in the *AcBb* co-culture than in either monoculture (*p* < 0.05). These findings demonstrate that co-cultivation enhanced butyrate production and provide experimental evidence consistent with a cooperative, cross-feeding-associated metabolic interaction between the two strains. Accordingly, TSB was selected for integration into the Mm 3D-HaeMan system.

We next tested host cell compatibility with these media by comparing TSB- and MRS-based conditions against CC without bacterial inoculation. Trypan blue staining showed that CCD841CoN cells maintained comparable viability under CC and TSB conditions, whereas viability in MRS was significantly reduced to 32.5% of that under CC (Figure 2a and b). Consistently, crystal violet staining revealed no notable morphological changes in CCD841CoN cells cultured in TSB within the Mm 3D-HaeMan system compared with that in CC, with similar cell density and structural integrity (Figure 2c and d). These results established TSB as the basal medium for subsequent co-culture experiments.

**Figure 2.**
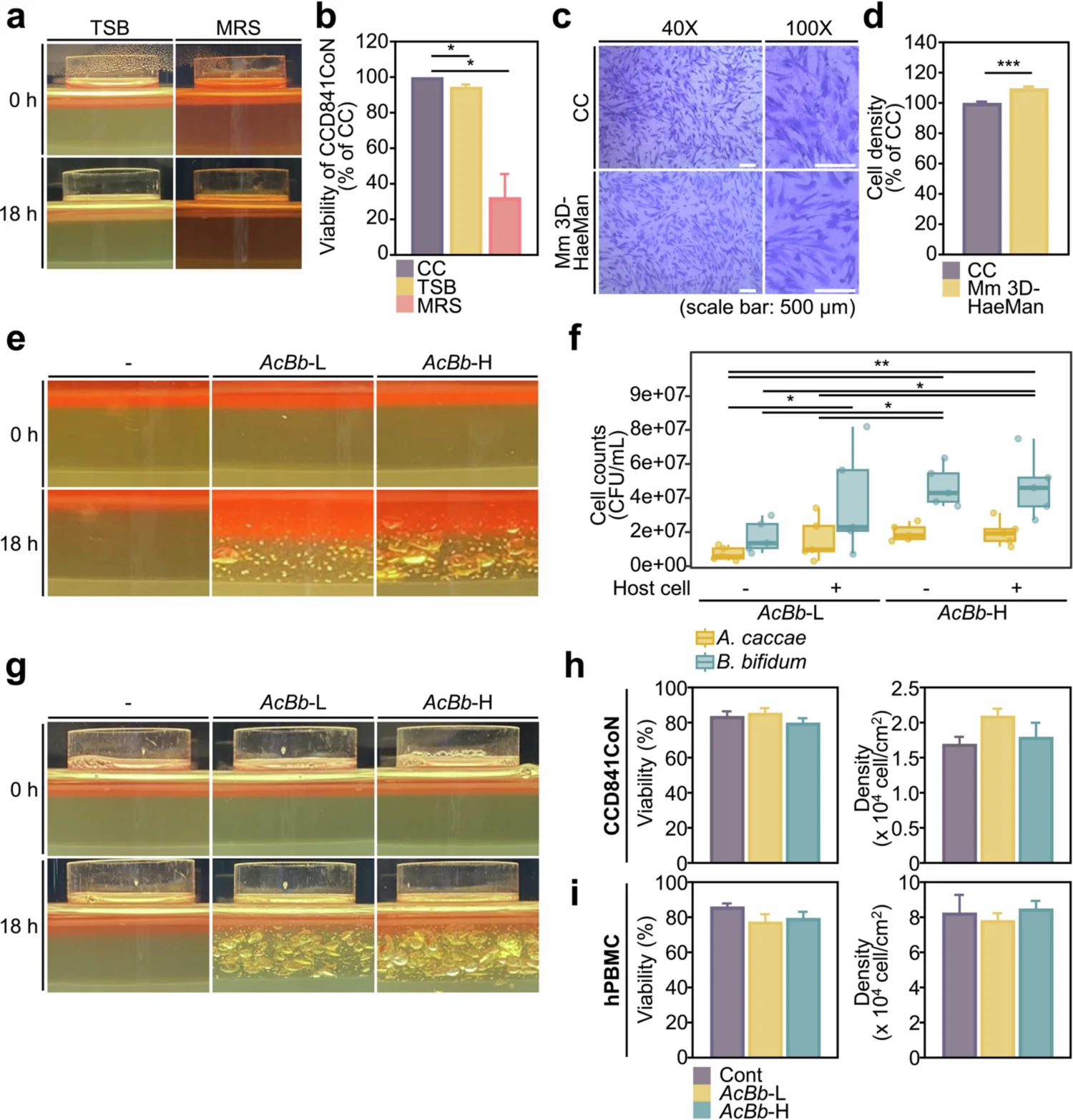
Optimization of co-culture conditions for short-chain fatty acids cross-feeding bacteria and human colon cells in the Mm 3D-HaeMan system. (a and b) Viability and density of CCD841CoN cells after 18 h of culture in the Mm 3D-HaeMan system containing either TSB- or MRS-based bacterial media, assessed by trypan blue exclusion. Data represent mean ± SD (*n* = 3). *, *p* < 0.05 vs. CC using Welch’s *t*-test. (c and d) Crystal violet staining of CCD841CoN cells cultured in the Mm 3D-HaeMan system (TSB) or under CC conditions. Quantification was performed by measuring the absorbance of ethanol-eluted dye. Data represent mean ± SD (*n* = 4). ***, *p* < 0.001 vs. CC using Welch’s *t*-test. (e) Growth of *Anaerostipes caccae* and *Bifidobacterium bifidum* (*AcBb*) in the Mm 3D-HaeMan system after 18 h at two inoculum levels: low (L; 250 cells of *A. caccae* + 2,500 cells of *B. bifidum*) and high (H; 500 cells of *A. caccae* + 5,000 cells of *B. bifidum*). (f) Abundance of *AcBb* in the semi-solid layer under varying host cell conditions and inoculum levels, measured by species-specific quantitative real-time polymerase chain reaction (qPCR). Data represent median ± SD (*n* = 5). *, *p* < 0.05; **, *p* < 0.01 using Dunn’s test with false discovery rate (FDR) correction. (g–i) Viability and density of CCD841CoN cells and hPBMCs following 18-h co-culture with low and high *AcBb* inocula. Data represent mean ± SD (*n* = 3). **, *p* < 0.01; ***, *p* < 0.001 vs. bacteria-free control using Welch’s *t*-test. CC, conventional culture.

To further optimize the co-culture parameters for the *A. caccae* and *B. bifidum* (*AcBb*), we tested multiple inoculum levels and incubation periods (Figure S2a–c). Excessive bacterial growth resulted in CO₂ accumulation and destabilization of the semi-solid Gelrite layer. Based on these evaluations, two inoculum levels were selected for the host co-culture experiments: a low condition (*AcBb*-L; *A. caccae* 250 cells and *B. bifidum* 2,500 cells in total) and a high condition (*AcBb*-H; *A. caccae* 500 cells and *B. bifidum* 5,000 cells in total), both of which maintained layer structural integrity over the 18 h co-culture period (Figure 2e). Unless otherwise stated, all subsequent co-culture experiments were performed using the *AcBb*-L inoculum. Species-specific qPCR indicated proliferation of both strains in the presence of host cells under both inoculum conditions (Figure 2f). Under these optimized co-culture conditions, no significant differences were observed in the viability or density of both CCD841CoN cells and hPBMCs compared with those of the *AcBb*-free controls (Figure 2g–i). Notably, the optimized system yielded sufficient biomass for downstream multi-omics, achieving over 2 × 10^7^ CFU/mL in the 40-mL bacterial compartment, 1.5 × 10^4^ cells/cm^2^ in the epithelial cell compartment, and 7.5 × 10^4^ cells/cm^2^ in hPBMCs.

To assess epithelial barrier function under Mm 3D-HaeMan co-culture conditions, TEER and V*te* were measured at 0 and 18 h across the CC, Cont, and *AcBb* groups (Figure S3a). TEER values were maintained in all three groups over the 18 h incubation period, with no statistically significant differences between conditions, providing no evidence that *AcBb* co-culture disrupted transepithelial resistance. V*te* increased significantly in both the Cont and *AcBb* groups between 0 and 18 h (both *p* < 0.001), whereas the CC group showed no significant change. At the 18 h endpoint, V*te* was significantly more negative in the *AcBb* group than in both the CC and Cont groups (both *p* < 0.05), indicating an *AcBb*-associated shift in transepithelial potential that may reflect changes in epithelial ion transport or the electrophysiological state within the Mm 3D-HaeMan system.

To further assess the junctional organization, immunofluorescence staining for ZO-1 was performed under the same conditions (Figure S3b). ZO-1 exhibited a similar distribution pattern across all three groups, with signals concentrated at cell–cell contacts. Z-stack imaging along the XZ axis confirmed that ZO-1 was localized preferentially toward the apical junctional region of the cell layer under all conditions, consistent with tight junction positioning. These observations indicate that neither the Mm 3D-HaeMan configuration nor *AcBb* co-culture overtly disrupted ZO-1 junctional localization relative to CC.

To assess whether the Mm 3D-HaeMan system was compatible with bacterial species beyond the *AcBb* consortium, we tested an additional cross-feeding pair, *F. prausnitzii* and *B. adolescentis* (*FpBa*), under the same configuration. Layer integrity showed no disruption, host cell viability and density were not significantly affected at lower inoculum levels, and both bacterial species were recovered from the bacterial compartment under host co-culture conditions (Figure S4a–c). These preliminary findings suggest that the system may be adaptable to microbial combinations beyond the *AcBb* consortium, although species-specific optimization of culture conditions is required for each new bacterial pair.

Collectively, these findings suggest that the Mm 3D-HaeMan system enables stable co-culture of strict anaerobes with host cells while maintaining epithelial viability and junctional organization, with no evidence of compromised transepithelial resistance under the experimental conditions tested. The maintained TEER values and apical ZO-1 localization, together with an *AcBb*-associated shift in V*te*, indicate that the system provides a functionally supportive setting for interrogating host–microbe interactions without disrupting epithelial integrity, thereby providing support for the downstream multi-omics analyzes described in subsequent sections.

### Host cell-specific transcriptional responses of SCFA cross-feeding bacteria in the Mm 3D-HaeMan system

After the establishment of optimized co-culture conditions, we next investigated how host cell presence modulates bacterial transcriptional responses within the Mm 3D-HaeMan system. Metatranscriptomic profiling was performed across four *AcBb*-containing conditions: bacteria cultured alone, bacteria co-cultured with CCD841CoN cells, with hPBMCs, or with both cell types (*n* = 3 per group). Total RNA and DNA were extracted from the bacterial compartment under each condition. Species-specific qPCR confirmed that the abundance of the *AcBb* consortium did not differ significantly across co-culture conditions (Figure 3a), indicating comparable bacterial growth across host contexts and minimizing bacterial load as a confounder for downstream transcriptional comparisons.

**Figure 3.**
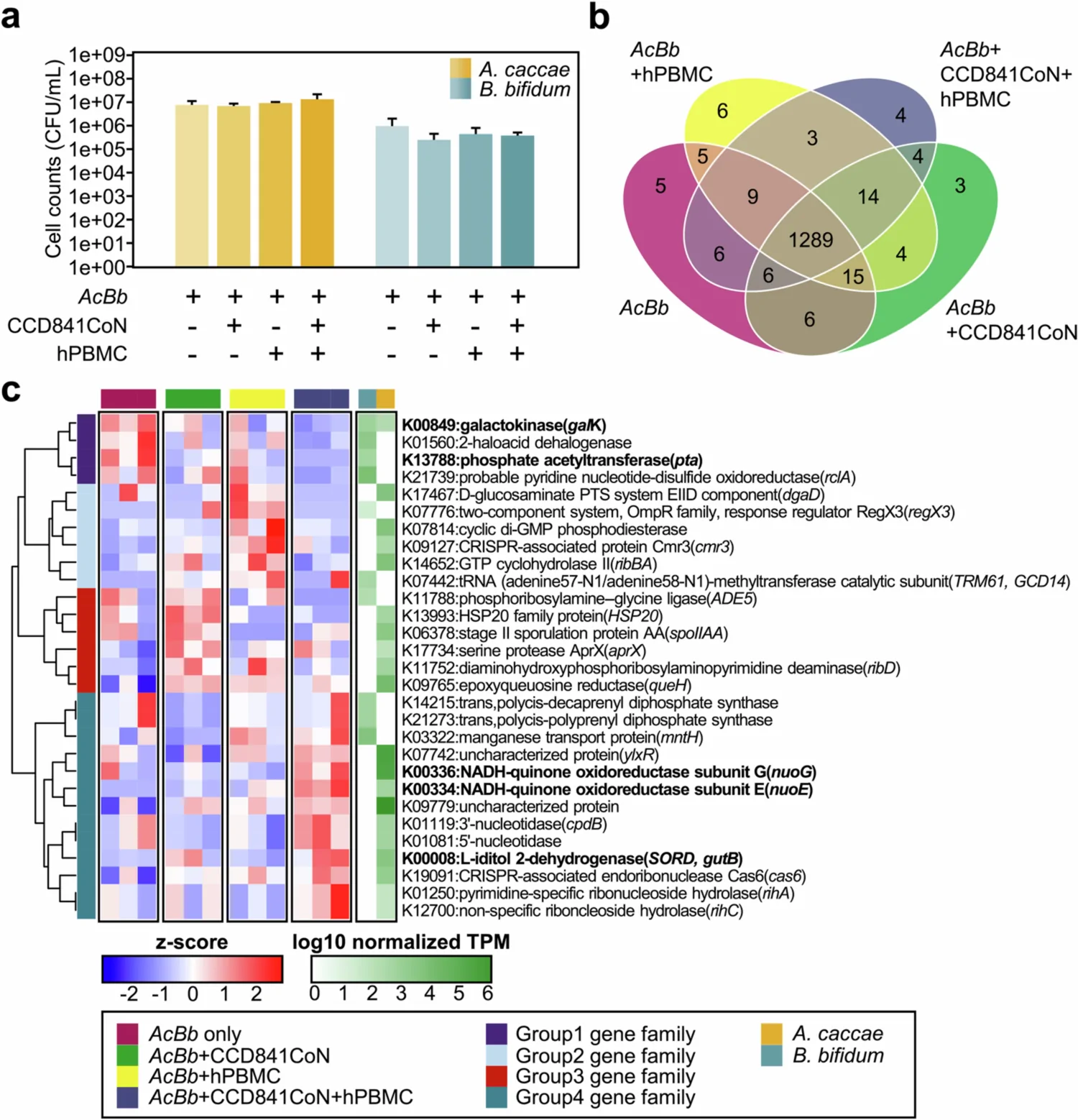
Metatranscriptomic profiling of SCFAs cross-feeders co-cultured with host cells in the Mm 3D-HaeMan system. (a) Quantification of *AcBb* abundance in the semi-solid layer under four co-culture conditions, assessed using species-specific qPCR. Bar plots show mean ± SD. Statistical analysis used Dunn′s test with Benjamini–Hochberg FDR correction. (b) Venn diagram showing shared and condition-specific bacterial transcripts across four co-culture conditions. (c) Heatmap of significantly altered bacterial transcripts (KEGG gene families, *n* = 3/group), clustering by Spearman correlation (tree height < 1.4). Gene families were identified using MaAsLin2 (adjusted *p* < 0.05, |coefficient| > 1). Log_10_-normalized TPM values represent contributions of *AcBb*; higher abundance is shown in green. TPM, transcripts per kilobase million.

To determine whether *AcBb* generates SCFA-related metabolites under the same experimental conditions used for the host response analyzes, we quantified SCFAs in the bacterial compartment of the Mm 3D-HaeMan system. Acetate, butyrate, propionate, and succinate were undetectable or below the detection limit under *AcBb*-free host cell control conditions. In contrast, these metabolites were detected after *AcBb* inoculation, supporting the establishment of a SCFA-containing metabolic environment within the bacterial compartment of the Mm 3D-HaeMan system by the *AcBb* consortium (Figure S5a). We further examined whether host cell-dependent co-culture conditions were associated with changes in SCFA-related bacterial metabolic activity. The pathway-level transcript abundances of butanoate metabolism (ko00650) and propanoate metabolism (ko00640) did not substantially differ across *AcBb*-containing co-culture conditions (Figure S5b). Consistently, SCFA concentrations did not differ significantly among bacteria cultured alone, co-cultured with CCD841CoN cells, hPBMCs, or both cell types. These findings indicated that *AcBb* generated SCFA-related metabolites within the Mm 3D-HaeMan system, whereas the host cell type did not substantially alter the overall SCFA output under the tested conditions.

Across all conditions, 1,289 transcripts were shared; however, each co-culture configuration also exhibited condition-specific transcriptional signatures (Figure 3b). Shared and condition-specific features were visualized using a Venn diagram. To identify significantly altered bacterial features across conditions, we performed multivariate association analysis using MaAsLin2-based pairwise comparisons among all bacterial cultures and host cell co-culture conditions with |coefficient| > 1 and *q* < 0.05 as significance thresholds (Figure S5c). This analysis identified 29 gene families with significant expression differences, which were clustered into four groups according to the co-culture conditions (Figure 4c). Group 1 comprised four gene families upregulated in bacteria-only culture; Group 2 included six gene families upregulated during co-culture with hPBMCs; Group 3 consisted of six gene families upregulated during co-culture with CCD841CoN cells; and Group 4 contained 13 gene families selectively upregulated during co-culture with both CCD841CoN cells and hPBMCs.

**Figure 4.**
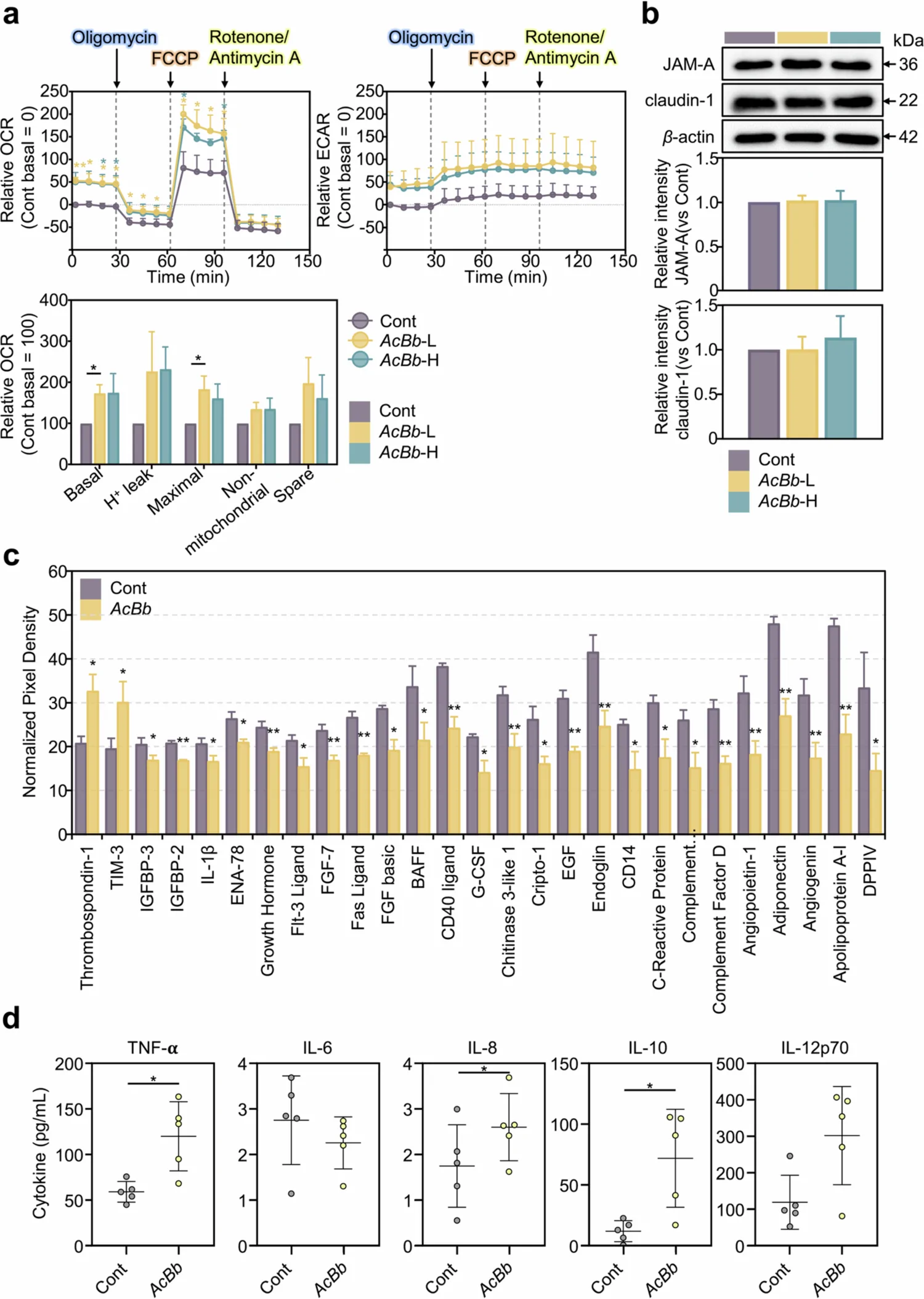
*AcBb*-mediated physiological and metabolic modulation in host epithelial and immune cells. (a) Mitochondrial respiration of CCD841CoN cells was analyzed using Seahorse XFp Mito Stress Test. Data represent mean ± SD (% of control basal, *n* = 3). *, *p* < 0.05; **, *p* < 0.01 vs. bacteria-free control using Welch’s *t*-test. (b) Expression of tight-junction proteins JAM-A and claudin-1 in CCD841CoN cells co-cultured with *AcBb*, normalized to *β*-actin. Data represent mean ± SD (*n* = 3). Welch’s *t*-test was used for comparisons. (c) Cytokine profiles of conditioned medium from CCD841CoN cells and hPBMCs co-cultured with *AcBb* (inoculum size: *AcBb*-L). Cytokines were measured using the Proteome Profiler Human XL Cytokine Array, and signal intensities were quantified by densitometry (duplicate spots). Pixel densities were normalized by cumulative sum scaling (CSS). Twenty-seven cytokines reached statistical significance (*p* < 0.05). (d) Quantitative cytokine profiling by cytometric bead array (CBA). Inflammatory cytokine levels in conditioned media from hPBMC cocultures in the presence or absence of *AcBb* were quantified using CBA. Statistical significance was evaluated using a *t*-test with Bonferroni correction. *, *p* < 0.05 vs. bacteria-free control. JAM, junctional-adhesion-molecule-A.

In the bacteria-only condition, the *glaK* gene, encoding a galactokinase that converts galactose to *α*-D-glucose-1-phosphate, was significantly upregulated compared with host cell-containing co-culture conditions. Conversely, during co-culture with both host cell types, *glaK* expression decreased, whereas *SORD*, encoding a sorbitol dehydrogenase that catalyzes the conversion of xylitol and NAD⁺ to D-xylulose and NADH, was upregulated compared with the bacteria-only condition. Furthermore, *nuoG* and *nuoE*, which encode the subunits of NADH-quinone oxidoreductase involved in NADH-linked electron transfer, were induced under these conditions. Because these functional assignments were based on gene annotation, we interpreted these changes cautiously as host-associated transcriptional changes in genes related to sugar alcohol utilization and NADH-linked energy metabolism, rather than as direct evidence of enhanced respiratory activity.

Among the 29 differentially expressed gene families, nine were assigned to *B. bifidum*, 19 to *A. caccae*, and one was shared between the two species. Notably, these co-culture condition-associated transcriptional differences occurred without detectable differences in bacterial abundance or overall SCFA output across *AcBb*-containing conditions, supporting niche-associated transcriptional regulation, which differences in bacterial abundance or SCFA levels alone may not fully explain. Collectively, these findings indicate that SCFA-cross-feeding bacteria generate an *AcBb*-derived SCFA-containing metabolic environment in the Mm 3D-HaeMan system and exhibit host cell-associated transcriptional responses that may reflect direct and indirect interactions with host epithelial and immune cells.

### AcBb-derived SCFA-producing co-culture conditions modulate epithelial mitochondrial function and immunoregulatory cytokine remodeling

To determine whether the host cells functionally responded to the *AcBb*-derived SCFA-containing metabolic environment established in the Mm 3D-HaeMan system, we assessed mitochondrial function, epithelial barrier integrity, and immune signaling in CCD841CoN cells co-cultured with *AcBb* (Figure 4). Mitochondrial respiratory function was evaluated using the Seahorse XFp Mito Stress Test, which measures OCR as a real-time indicator of mitochondrial oxidative phosphorylation and ECAR as a proxy for glycolytic activity.^48^ ^,^ ^49^ Co-culture with *AcBb* significantly increased both OCR and ECAR in CCD841CoN cells compared to those in bacteria-free controls across multiple parameters, including basal respiration, proton leakage, and maximal respiratory capacity, consistent with the increased bioenergetic demand and metabolic activity in CCD841CoN cells under these conditions (Figure 4a).

To further examine whether the cellular metabolic responses observed in CCD841CoN cells depended on the presence of viable bacteria or could be recapitulated by heat-killed preparations or cell-free factors, the cells were exposed to live bacteria, heat-killed bacteria, and culture supernatants from *A. caccae* alone, *B. bifidum* alone, and the *AcBb* consortium under equivalent inoculum conditions (Figure S6). The greatest increase in relative MTT activity was observed with live *AcBb* (*p* < 0.05), whereas the heat-killed preparations and culture supernatants did not replicate this effect under the tested conditions. Live *A. caccae* alone also showed a significant increase, although to a lesser extent than the consortium. These findings suggest that exposure to viable bacteria contributes to the increased MTT signal observed under Mm 3D-HaeMan conditions.

To assess epithelial barrier integrity under *AcBb* co-culture conditions, expression of the tight junction-associated proteins JAM-A and claudin-1 was evaluated by Western blot analysis. Neither protein showed significant changes in expression following *AcBb* co-culture relative to bacteria-free controls, indicating that these junctional components were not disrupted at the protein level (Figure 4b). These findings are consistent with the functional barrier data presented in Figure S3a, suggesting that the *AcBb*-derived metabolic environment did not compromise the epithelial barrier function under the present experimental conditions.

Analysis of the conditioned medium from the host compartment further revealed selective remodeling of secreted immune mediators in response to *AcBb*. Specifically, thrombospondin-1 (TSP-1) and T-cell immunoglobulin and mucin domain-containing protein 3 (TIM-3) were significantly upregulated, whereas multiple complement, chemotactic, angiogenic, proliferation-related, and inflammatory mediators-including IL-1*β*, ENA-78, CD40L, G-CSF, CHI3L1, CRP, complement component C5/C5a, CFD and DPP-4, IGFBP-2, IGFBP-3, growth hormone, FGF-7, basic FGF, BAFF, Crypto-1, EGF, endoglin, angiopoietin-1, adiponectin, angiogenin, and apolipoprotein A-I, Flt-3 ligand, FasL, and CD14-were significantly downregulated (Figure 4c). The upregulation of TSP-1 and TIM-3 is consistent with their established roles in activating latent TGF-*β* and constraining Th1 immune activation, respectively,^50^ ^,^ ^51^ whereas the coordinated reduction in complement, chemotactic, and angiogenesis-related mediators suggest broad remodeling of immune signaling pathways. However, in the absence of a direct *in vivo* comparator, these directional changes should be regarded as hypothesis-generating observations rather than a demonstrated shift toward a defined immunoregulatory state.

To complement the semi-quantitative cytokine array, we performed quantitative cytokine measurements using the BD Human Inflammatory Cytokine CBA kit. Quantitative analysis revealed that co-culture with the *AcBb* consortium resulted in significantly increased concentrations of TNF-*α*, IL-8, and IL-10 in the conditioned medium, while IL-6 remained unchanged relative to bacteria-free controls (Figure 4d).

Collectively, these results indicate that *AcBb*-derived SCFA-producing co-culture conditions are associated with increased epithelial bioenergetic parameters, no overt disruption of barrier-related readouts, and context-dependent cytokine remodeling involving concurrent modulation of both pro- and anti-inflammatory mediators, supporting the utility of the Mm 3D-HaeMan system for the controlled interrogation of host–microbe interactions under colon-like conditions.

### Host epithelial transcriptome remodeling in response to SCFA cross-feeding bacteria in the Mm 3D-HaeMan system

To further elucidate molecular mechanisms underlying the host responses observed in the Mm 3D-HaeMan system, we profiled the transcriptome of CCD841CoN cells after co-culture with the *AcBb* consortium. PCA showed a substantial overlap between the transcriptomes of *AcBb*-co-cultured cells and those of bacteria-free controls, indicating that *AcBb* did not induce a strong global shift in the epithelial transcriptome under these conditions (Figure 5a). Nevertheless, differential expression analysis identified 41 significantly altered genes (|log_2_ FC| > 1, *p* < 0.05), comprising 15 upregulated and 26 downregulated transcripts among 5,566 expressed genes (Figure 5b).

**Figure 5.**
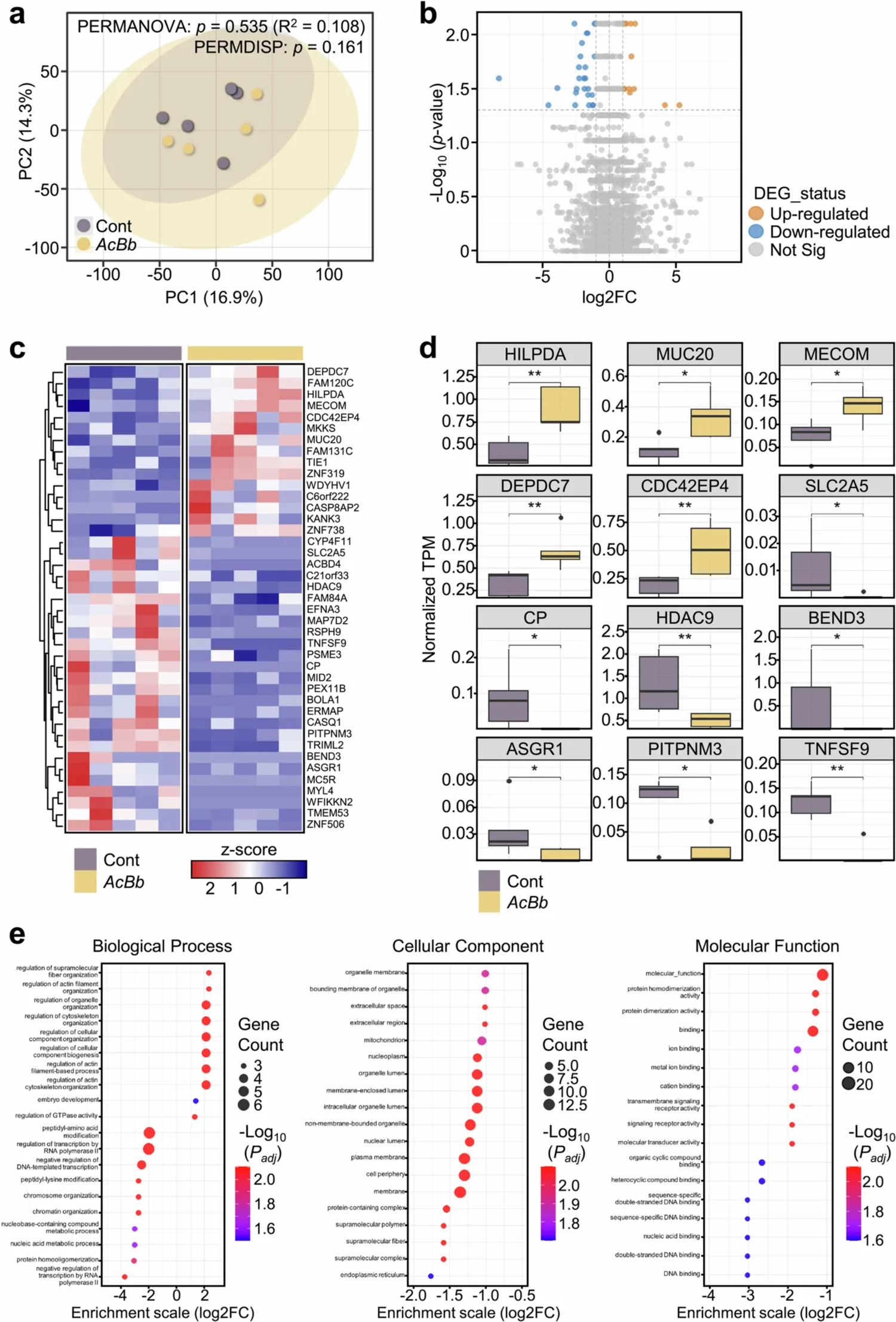
Transcriptome remodeling of CCD841CoN cells induced by SCFA cross-feeders in the Mm 3D-HaeMan system. (a) Principal-component-analysis (PCA) of transcriptomes from CCD841CoN cells co-cultured with *AcBb* (red) or maintained in control conditions (Cont; blue). Group separation was assessed by PERMANOVA (Euclidean distance on centered and scaled data, permutations = 999, *n* = 5/group), reporting *p*-value and R^2^. Homogeneity of dispersion was tested using PERMDISP. (b) Volcano plot showing differential expression between *AcBb*-co-cultured and control CCD841CoN cells (*n* = 5/group). Orange: upregulated DEGs (*n* = 15); blue: downregulated DEGs (*n* = 26); gray: non-significant genes (*n* = 5,525). (c) Heatmap of DEGs (CSS-normalized TPM; z-score transformed) with hierarchical clustering (*n* = 5/group). (d) Expression levels of DEGs associated with cellular energy metabolism, intestinal barrier function, and cell signaling pathways. Data are CSS-normalized TPM values. Wilcoxon rank-sum test with Benjamini–Hochberg FDR correction was applied. *, *p* < 0.05; **, *p* < 0.01 vs. bacteria-free control (*n* = 5/group). (e) GO enrichment analysis of DEGs from *AcBb* co-cultured CCD841CoN cells. The top 10 enriched and bottom 10 depleted GO terms in Biological Process, Cellular Component, and Molecular Function categories are shown. Dot size indicates gene count; dot color indicates –log_10_ adjusted *p*-value.

Functional annotation of differentially expressed genes (DEGs) revealed coherent biological themes (Figure 5c and d). The gene names used in this section are defined in the abbreviation list. Among the upregulated genes, HILPDA is associated with lipid metabolism under hypoxic and metabolically stressed conditions.^52^ MUC20 is also associated with mitochondrial calcium homeostasis and epithelial integrity.^53^ ^,^ ^54^ MECOM encodes a transcriptional regulator implicated in epithelial stress response and cell survival.^55^ ^,^ ^56^ MUC20 and CDC42EP4 are associated with epithelial polarity and junctional regulation,^53^ whereas DEPDC7 is associated with apoptosis regulation in other epithelial contexts; however, their^57^ relevance to the present system is inferential. However, the functional roles of these genes in the present co-culture context remain to be directly established, and their inclusion here is intended to be hypothesis-generating rather than mechanistically conclusive. Among the downregulated genes, SLC2A5, a fructose transporter, is implicated in intestinal nutrient absorption.^58^ CP is associated with macrophage-mediated inflammatory responses in patients with IBD.^59^ HDAC9 is elevated in the inflamed intestinal mucosa,^60^ and its activity is suppressed by butyrate.^61^ TNFSF9 has also been implicated in macrophage inflammatory signaling.^62^ BEND3, ASGR1, and PITPNM3, whose roles in the present co-culture context remain to be established, are directionally consistent with reduced inflammatory activation and altered nutrient utilization. However, these associations were inferred from functional annotations in other contexts and require orthogonal validation in the present system.

GO enrichment analysis corroborated these trends (Figure 5e). In the Biological Process category, DEGs were enriched for regulation of actin filament organization (GO:0110053; adjusted *p* < 0.01) and organelle organization (GO:0033043; adjusted *p* < 0.01), consistent with cytoskeletal and mitochondrial remodeling. Conversely, negatively enriched terms included negative regulation of transcription by RNA polymerase II (GO:0000122; adjusted *p* < 0.01), chromatin organization (GO:0006325; adjusted *p* < 0.01), and nucleic acid metabolic process (GO:0090304; adjusted *p* < 0.05), suggesting attenuation of broad transcriptional activation programs. Decreased enrichment of mitochondrion- and membrane-associated cellular components, together with depletion of DNA-binding and signaling receptor activity terms, further supported reduced transcriptional and receptor-driven activation. Collectively, these results indicate that *AcBb* co-culture elicited a modest but statistically defined transcriptional response, with DEGs nominally associated with metabolic and cytoskeletal processes. The direction of these changes broadly concords with the functional readouts observed in the mitochondrial and cytokine assays, although the transcriptional data alone are insufficient to establish mechanistic causality and should be interpreted as hypothesis-generation. Orthogonal validation, including the protein-level quantification of selected DEG products or functional perturbation experiments, is required to establish the mechanistic significance of individual transcriptional changes.

## Discussion

In this study, we developed and validated the Mm 3D-HaeMan system, a vertically stratified host-microbe co-culture platform that recapitulates the key physiological features of the human colon, including stable oxygen gradients; spatial separation of epithelial, immune, mucus, and microbial layers; and compatibility with strict anaerobes (Figure 1). Using this system, we established controlled co-cultures of normal human colonic epithelial cells, hPBMCs, and a SCFA cross-feeding consortium (*A. caccae* and *B. bifidum*), enabling systematic interrogation of bacterial growth, metabolic functions, and host responses. Importantly, the compartmentalized architecture permits the independent sampling of microbial, epithelial, and culture-medium layers without disturbing overall structural integrity, enabling layer-resolved analyzes—including microbial meta-omics, epithelial transcriptomics and functional assays, and cytokine or metabolite profiling—thereby supporting multidimensional mechanistic studies. Collectively, these capabilities support Mm 3D-HaeMan as a controlled and analytically integrated platform for investigating bidirectional host–microbe interactions under controlled, colon-like conditions.

Oxygen profiling demonstrated that each compartment of the Mm 3D-HaeMan system achieved oxygen tensions within the ranges reported for the human colon (Figure 1b), where the luminal pO₂ was less than 1 mmHg (equal. < 0.13%) in the cecum, and approximately 3 mmHg (equal. ~ 0.4%) in the distal colon.^63^ Since oxygen is a dominant ecological driver of gut microbial structure and function, accurately reproducing these gradients is a foundational requirement for modeling human intestinal physiology. The ability to maintain oxygenated conditions for host cells while approaching near-anoxic conditions in the microbial compartment is particularly important for sustaining strict anaerobes and interpreting metabolite-driven host responses in a physiologically meaningful context.

To provide an epithelial component with greater transcriptional and metabolic proximity to normal colonic tissue than to carcinoma-derived lines, we used the non-tumorigenic human colonic epithelial cell line CCD841CoN rather than carcinoma-derived Caco-2 cells, which can exhibit altered metabolic programs, abnormal proliferation, and small-intestinal differentiation biases that confound the interpretation of microbial and metabolic effects.^64^ The CCD841CoN cells retained stable viability, morphology, tight-junction structure, and mitochondrial responsiveness during co-culture, indicating that the system provides a supportive and non-disruptive environment for epithelial function. This approach also provides a pathway toward customized *ex vivo* extensions using patient- or animal-derived epithelial cells to further enhance physiological relevance. However, we acknowledge that CCD841CoN cells do not form a fully polarized columnar monolayer equivalent to the primary colonic epithelium and that their flattened morphology under standard culture conditions is consistent with the ATCC annotation that definitive evidence of epithelial origin is lacking. Despite these morphological limitations, functional assessment confirmed the measurable transepithelial electrical resistance and preferential apical localization of ZO-1, indicating that the cells retained their functional junctional properties under the present co-culture conditions (Figure S3a and S3b). Therefore, the use of CCD841CoN should be understood within the context of a reductionist experimental design that prioritizes accessibility and reproducibility over full recapitulation of the colonic epithelial architecture.

The inclusion of hPBMCs provided an experimentally tractable approach for modeling human immune responses, consistent with their widespread use in studies of cytokine and chemokine signaling, inflammatory cascade activation, and metabolite-mediated immune modulation.^65^ ^,^ ^66^ Prior work indicates that PBMC co-culture can capture the metabolite-dependent regulation of epithelial barrier function and immune signaling,^65^ including pathways relevant to regulatory T (T_reg_) cell activation and T-helper 17 (Th17) suppression, and innate immune responses.^66^ ^,^ ^67^ In the present study, hPBMCs enabled an integrated assessment of epithelial–immune crosstalk in response to microbial metabolism and co-culture-derived signals (Figure 4c and d).

To sustain the commensal growth of the SCFA cross-feeding pair *AcBb*, we systematically evaluated candidate culture media under matched inoculum densities, oxygen tensions, and incubation times (Figure S1a–c). TSB supported sufficient growth of both species and yielded the highest acetate and butyrate production while maintaining epithelial viability, in contrast to MRS, which, due to its polysorbate 80 content, has been reported to impair epithelial integrity and mitochondrial function.^68^ These findings highlight the underappreciated role of media formulation in balancing host–microbe coexistence and underscore the value of gut-mimetic or chemically defined media, which can better approximate *in vivo* nutrient landscapes and microbial metabolic outputs.^69-71^

To evaluate whether the Mm 3D-HaeMan configuration maintained epithelial barrier function during co-culture, TEER and V*te* were measured under CC, Cont, and *AcBb*-co-culture conditions (Figure S3a). The TEER values were stable across all groups over the co-culture period, but V*te* increased significantly in both the Cont and *AcBb* groups, with the *AcBb* group showing a more negative potential at 18 h relative to the CC and bacteria-free controls. These findings provide functional evidence that the Mm 3D-HaeMan system does not compromise epithelial barrier competence.

Compared with earlier SCFA cross-feeder studies that primarily quantified metabolites and assessed barrier parameters,^72-75^ the Mm 3D-HaeMan enables integrated, multilayer analysis within a single experimental framework—including epithelial bioenergetics (OCR/ECAR), tight-junction protein expression, multiplex immune profiling, and combined host and bacterial transcriptomics. This integrated design enabled us to observe responses consistent with the previously reported effects of SCFA cross-feeding and explore pathway-level associations at the host–microbe interface.

Consistent with prior studies reporting that acetate-to-butyrate cross-feeding can enhance oxidative metabolism and reduce pro-inflammatory signaling in colonic models,^73^ ^,^ ^76^ the *AcBb* co-culture was associated with higher mitochondrial respiration parameters in CCD841CoN cells (Figure 4a). This was reflected by elevated basal OCR, proton leak, and maximal respiratory capacity, together with an increase in ECAR. The parallel elevation of OCR and ECAR suggests a coordinated energetic shift that may integrate oxidative phosphorylation with glycolytic support for biosynthetic demands.^48^ In support of the use of the *AcBb* consortium as the primary microbial condition in this study, a comparative MTT-based assessment further showed that exposure to live *AcBb* produced the largest increase in cellular reductive activity relative to bacteria-free controls and was greater than that observed with either species alone (Figure S6). In contrast, heat-killed preparations and cell-free supernatants did not recapitulate the live *AcBb*-associated increase under the tested conditions. Although MTT reflects global cellular reductive activity rather than a mitochondria-specific mechanism, these comparative controls suggest that the consortium context provides a more pronounced host metabolic stimulus than single-species exposure, supporting the use of *AcBb* as the principal condition for downstream functional and multi-omics analyzes within a co-culture framework.

*AcBb* co-culture did not reduce JAM-A or claudin-1 protein expression, indicating that these junctional components were not overtly disrupted at the protein level, which is consistent with the functional barrier data presented above (Figure 4b). Importantly, because junctional protein abundance alone does not establish barrier function or correct subcellular localization, we interpreted these immunoblot results in the context of additional readouts. In particular, the stability of TEER over the 18 h co-culture and the preserved apical-junctional localization of ZO-1 by immunofluorescence together provided functional and spatial support for the conclusion that the epithelial layer did not show evidence of acute barrier compromise under these conditions (Figure S3).

*AcBb* co-culture was associated with a broad shift in cytokine profiles, including increased TSP-1 and TIM-3 levels and decreased levels of multiple mediators linked to inflammatory, complement, chemotactic, and angiogenic processes (Figure 4c).^50^ ^,^ ^51^ These findings align with the established roles of TSP-1 in activating latent TGF-*β* and promoting mucosal restitution,^77^ and TIM-3 in constraining Th1 and innate immune activation,^50^ although causal roles were not directly tested in our system. Likewise, the observed reductions in C5/C5a, ENA-78, Flt-3 ligand, and CD14 may be compatible with dampened inflammation-associated signaling, but should be interpreted cautiously given the *in vitro* setting and the inclusion of PBMCs as a major source of secreted factors.^78-80^

Quantitative cytokine profiling by CBA further demonstrated significant elevation of TNF-*α*, IL-8, and IL-10 under *AcBb* co-culture conditions, while IL-6 remained unchanged (Figure 4d). The concurrent increase in both pro-inflammatory (TNF-*α*, IL-8) and anti-inflammatory (IL-10) mediators suggests a context-dependent modulation of the cytokine milieu rather than a uniformly suppressive response. The pattern of concurrent pro- and anti-inflammatory cytokine elevation observed here differs from the predominantly suppressive cytokine profiles reported in prior studies on Bifidobacterium conducted under pre-inflammatory or stimulated conditions,^81^ suggesting that the cytokine response in the present system may reflect a qualitatively different immune context rather than a failure to recapitulate anti-inflammatory effects.

Collectively, these data indicate that *AcBb* co-culture is associated with cytokine remodeling, including the concurrent modulation of both pro- and anti-inflammatory mediators (Figure 4c and d). Although these patterns are compatible with known SCFA-responsive regulatory pathways, additional pathway-level validation is required to link these cytokine changes directly to SCFA-mediated GPCR signaling or HDAC inhibition.^82-84^

Host transcriptomics provided additional directional information (Figure 5). The upregulation of *HILPDA*, *MUC20*, and *MECOM* is consistent with the activation of programs supporting hypoxia adaptation, epithelial remodeling, and mitochondrial metabolism,^52-56^ whereas the downregulation of *SLC2A5*, *CP*, *HDAC9*, *BEND3*, and *TNFSF9* aligns with reduced inflammatory activation and altered nutrient utilization (Figure 5c and d).^58-62^ ^,^ ^85^ GO enrichment analysis identified actin filaments and organelle organization as the most significantly enriched biological processes among the upregulated DEGs (Figure 5e). Although these terms are nominally consistent with the mitochondrial functional changes observed in the Seahorse assay, the transcriptional data do not directly demonstrate cytoskeletal–mitochondrial coupling in this system, and this interpretation should be regarded as hypothesis-generating rather than mechanistically established.^53^ ^,^ ^86^ Together, the transcriptional data provide a modest, hypothesis-generating signal that is directionally consistent with functional and cytokine readouts but should not be interpreted as independent mechanistic evidence for these phenotypes. Orthogonal validation, such as protein-level quantification of selected DEG products or functional perturbation experiments, is required to establish the biological significance of individual transcriptional changes.

Metatranscriptomic analysis revealed reciprocal bacterial adaptation to the presence of host cells. *A. caccae* and *B. bifidum* remodeled metabolic gene expression in the presence of epithelial or immune cells, shifting substrates and energy pathways in a host cell-context-dependent manner (Figures 3a–c and S5c). These host-influenced transcriptional programs reflect known principles of bacterial resource partitioning, cross-feeding adaptability, and microbe–host metabolic interactions.^87^ ^,^ ^88^

In conclusion, these findings indicate that *AcBb* co-culture under SCFA-producing conditions is associated with changes in epithelial metabolic activity, junctional protein expression, cytokine profiles, and host cell context-dependent bacterial transcriptional responses. The Mm 3D-HaeMan system therefore represents a controlled and analytically accessible platform for interrogating reciprocal host–microbe dynamics at the mucosal interface under defined oxygen gradients.

Several limitations should be considered. First, although CCD841CoN cells were selected as a non-tumorigenic human colonic epithelial cell line, the relatively low TEER values and non-uniform ZO-1 staining pattern indicate that this cell layer does not constitute a high-resistance, fully polarized intestinal epithelial barrier comparable to differentiated Caco-2 monolayers, primary intestinal epithelium, or organoid-derived epithelial models (Figure S3).^89^ These characteristics reflect the intrinsic limitations of the current CCD841CoN-based monolayer configuration as a mature barrier model. Therefore, the TEER, V*te*, ZO-1, JAM-A, and claudin-1 data should be interpreted as barrier integrity-related readouts indicating no overt epithelial deterioration under the tested conditions, rather than as evidence of complete physiological barrier function (Figure S3 and 4b). Future studies using primary colonic epithelial cells or organoid-derived epithelial layers will further improve the physiological barrier fidelity of this platform. Nevertheless, the present model provides a reproducible oxygen-stratified platform for mechanistic investigation of host–microbe interactions under controlled experimental conditions.

Second, although *AcBb* inoculation established an SCFA-containing metabolic environment in the Mm 3D-HaeMan system (Figure S5a), the present study did not establish whether SCFAs alone are exclusive drivers of the observed host phenotypes. While culture supernatants did not reproduce the metabolic response observed with live bacterial fractions under the tested conditions (Figure S6), this does not exclude the contributory role of secreted metabolites under the full co-culture configuration; bacterial structural components, direct cell-associated signals, or other interactions may also contribute to the observed host responses. Therefore, the mitochondrial, barrier-related, and immunoregulatory responses observed here should be interpreted as host responses associated with *AcBb*-derived conditions, rather than as effects attributable to any single bacterial component. Future perturbation experiments using defined SCFA supplementation and SCFA receptor inhibition are needed to resolve SCFA-specific causality.

Third, because the cytokine antibody array provides a semi-quantitative survey of a broad panel of secreted proteins, whereas the CBA quantitatively measures a limited set of inflammatory cytokines, these approaches provide complementary rather than directly comparable information. Therefore, the cytokine profiling results should be interpreted in light of the methodological differences between the two analytical platforms (Figure 4c and d). The cytokine array serves as a broad screening platform for global immune-response profiling, whereas the CBA assay provides targeted quantitative assessment of selected inflammatory cytokines. Accordingly, complete concordance between the two platforms is not necessarily expected because of differences in detection principle, antibody characteristics, analytical sensitivity, dynamic range, normalization strategy, and analyte coverage. In the present study, the two assays showed partial agreement rather than contradictory results. TNF-*α* exhibited a similar increasing trend in both platforms, showing an approximately 1.5-fold increase in the cytokine array (Dataset 4), although statistical significance was achieved only in the CBA assay. In contrast, statistically significant increases in IL-8 and IL-10 were detected only by the CBA assay (Figure 4d), likely reflecting its greater quantitative sensitivity for these selected cytokines rather than biologically contradictory responses. Taken together, the cytokine array and CBA assay should be regarded as complementary analytical approaches that together provide a more comprehensive characterization of the immune response than either platform alone. Accordingly, the combined cytokine profile should not be interpreted as evidence of a uniformly pro-inflammatory or anti-inflammatory state but rather as context-dependent cytokine remodeling involving both inflammatory and regulatory mediators. Future orthogonal validation using targeted quantitative approaches, such as ELIZA or additional bead-based immunoassays, will further strengthen the interpretation of cytokine responses observed in the Mm 3D-HaeMan system.

The current configuration relies on a simplified arrangement of barrier-constituting cells and a defined dual-strain consortium, thereby it does not fully capture the cellular heterogeneity of the intestinal mucosa or the taxonomic and functional diversity of the native gut microbiota.^90^ ^,^ ^91^ In particular, hPBMCs provide an experimentally tractable immune compartment, consistent with their use in epithelial–immune co-culture and SCFA-response studies, but they represent a circulating immune fraction rather than gut-resident mucosal immune populations.^65^ ^,^ ^66^ Future iterations incorporating tissue-relevant immune subsets may better capture mucosal immune regulation, given the diversity and regional specialization of immune populations in the human intestinal tract.^92^

Additionally, the immobilization of bacteria within the semi-solid Gelrite matrix can limit direct physical contact between bacterial cells and between bacteria and the host epithelium. This design helps maintain a stable oxygen gradient and minimize bacterial overgrowth or encroachment into the epithelial compartment. This also means that the system is particularly well-suited for investigating metabolite-mediated host–microbe interactions, rather than contact-dependent processes such as direct bacterial adhesion, biofilm formation, or effector translocation.

The preliminary compatibility of the Mm 3D-HaeMan system with the *FpBa* pair suggests that the platform is adaptable to microbial combinations beyond the *AcBb* consortium (Figure S4). However, each new bacterial combination will likely require species-specific optimization of medium composition, inoculum density, and incubation conditions, reflecting inherent differences in growth requirements and interspecies interaction dynamics. Once optimized, the core system architecture, including the co-culture period and oxygen-stratified compartment design, can be consistently applied across combinations. Similarly, the epithelial component is not restricted to CCD841CoN cells. Alternative cell lines or patient-derived epithelial cells can, in principle, be substituted and subjected to compatibility testing. Future iterations incorporating additional epithelial lineages and tissue-relevant immune subsets, as well as high-complexity consortia, will further improve physiological fidelity and broaden applicability across biological contexts.

Finally, the Mm 3D-HaeMan system enables three main translational applications (Figure 6). First, it enables the investigation of gut receptor-mediated host–microbe interactions and the functional evaluation of food-derived substances or candidate microbiome therapeutics, leveraging integrated bioenergetics, multiplex immune profiling, and multi-omics. Second, its workflow is compatible with personalization, enabling the incorporation of patient-derived epithelial cells and microbiota to model inter-individual or disease-specific variations. Third, its stratified compartments generate sufficient biomass for large-scale meta-omics, supporting the screening of next-generation probiotics, defined microbial consortia, and postbiotics. Collectively, these features position the Mm 3D-HaeMan system as a step toward bridging reductionist *in vitro* approaches and translational microbiome research.

**Figure 6.**
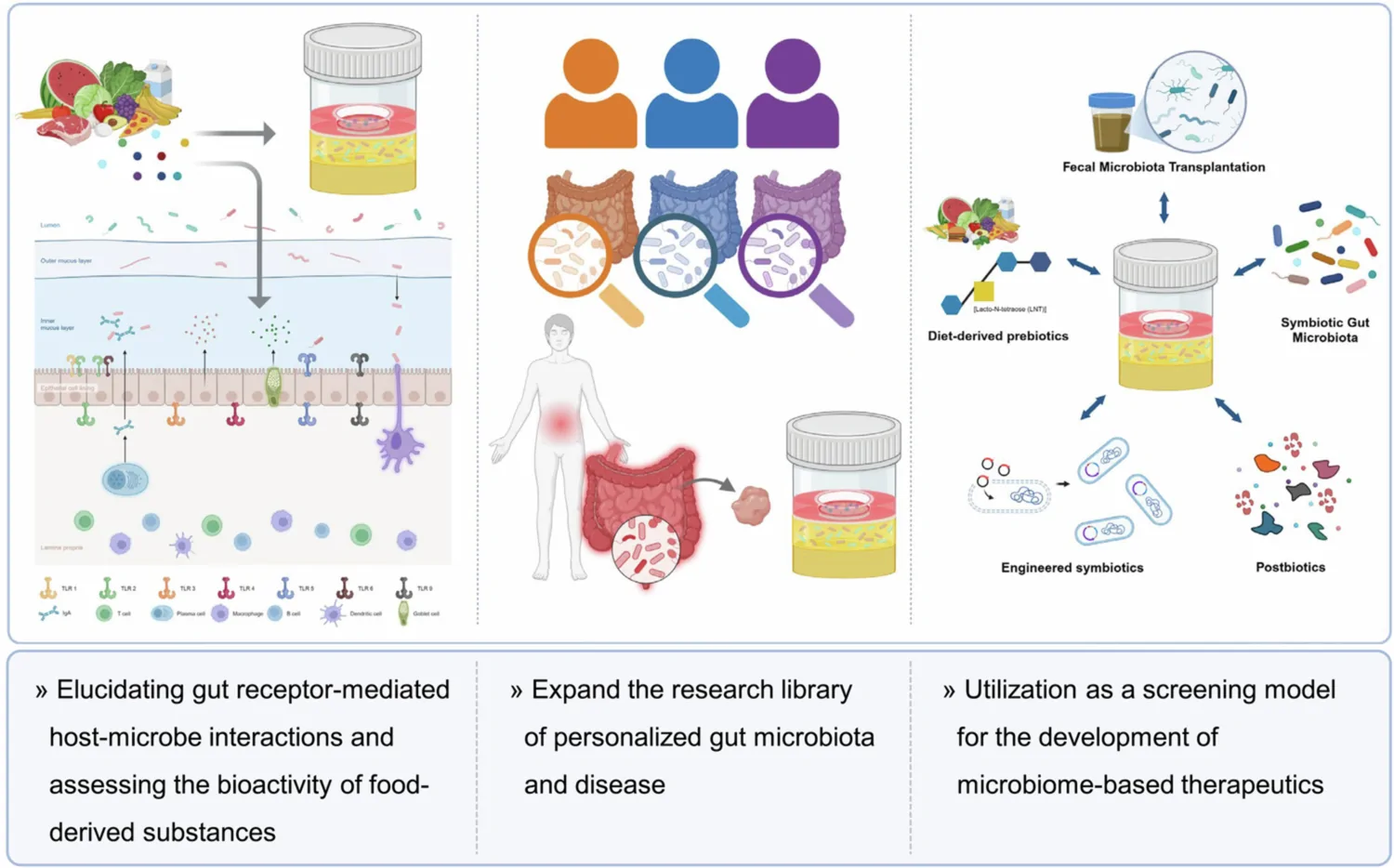
Translational significance and potential applications of the Mm 3D-HaeMan system. (i) Mechanistic studies of gut receptor-mediated host–microbe interactions and functional evaluation of food-derived bioactive molecules. Dietary components introduced into the system can be examined in the context of the mucosal interface, in which Toll-like receptors (TLR1–TLR6 and TLR9), secretory IgA, and lamina propria immune cells (T cells, B cells, plasma cells, macrophages, dendritic cells) together with goblet cells mediate host responses across the outer and inner mucus layers and the epithelial lining. (ii) Personalized modeling of host–microbiota interactions using donor- or patient-derived microbiota and epithelial cells, enabling inter-individual differences in microbial composition and disease-associated microbiota to be reconstructed *in vitro*. (iii) Screening platform for the development of microbiome-based therapeutics, including fecal microbiota transplantation, diet-derived prebiotics such as lacto-N-tetraose, symbiotic gut microbiota, engineered symbionts, and postbiotics.

## Supplementary Material

Figure S2.jpg Figure S2.jpg

Figure S4.jpg Figure S4.jpg

Figure S6.jpg Figure S6.jpg

Figure S5.jpg Figure S5.jpg

Figure S1.jpg Figure S1.jpg

Figure S3.jpg Figure S3.jpg

Clean copy_2nd_Rev_Supplementary_Materials_260720.docx Clean copy_2nd_Rev_Supplementary_Materials_260720.docx

## Data Availability

Sequence data generated in this study have been deposited in the European Nucleotide Archive (ENA) at EMBL-EBI under study number PRJEB97442. (https://ebi.ac.uk/ena/browser/view/PRJEB97442).

## Abbreviations

*AcBb*: *Anaerostipes caccae* and *Bifidobacterium bifidum*
ANOVA: Analysis of Variance
ASGR1: asialoglycoprotein receptor 1
BAFF: B-cell activating factor
BH: Benjamini–Hochberg
BEND3: BEN domain containing 3
CAD: Collision gas
CDC42EP4: Cell division cycle 42 effector protein 4
CD40L: CD40 ligand
CFD: Complement factor D
CFU: Colony forming unit
CHI3L1: Chitinase 3-like 1
CP: Ceruloplasmin
CRP: C-reactive protein
CSS: Cumulative sum scaling
Ct: Cycle threshold
DPP-4: Dipeptidyl peptidase-4
DEG: Differentially expressed gene
DEPDC7: DEP domain containing 7
ECAR: Extracellular acidification rate
EGF: Epidermal growth factor
ENA-78: Epithelial neutrophil-activating peptide 78
EMEM: Eagle’s minimum essential medium
FasL: Fas ligand
FBS: Fetal bovine serum
FCCP: Carbonyl cyanide-4-(trifluoromethoxy) phenylhydrazone
FDR: false discovery rate
FGF: Fibroblast growth factor
Flt-3: Fms related receptor tyrosine kinase 3
G-CSF: Granulocyte colony-stimulating factor
GO: Gene Ontology
hPBMCs: Human peripheral blood mononuclear cells
HDAC9: Histone deacetylase 9
HILPDA: Hypoxia-inducible lipid droplet-associated protein
IGFBP: Insulin-like growth factor-binding protein
IL: Interleukin
Insert: SPLInsert™ Standing
JAM-A: Junctional-adhesion-molecule-A
KEGG: Kyoto Encyclopedia of Genes and Genomes
KOs: KEGG Orthologs
MaAsLin2: Multivariate association with linear models 2
MECOM: MDS1 and EVI1 complex locus
MEM: Minimum essential medium
MRM: Multiple reaction monitoring
MRS: de Man, Rogosa and Sharpe medium
MTT: 3-(4,5-dimethylthiazol-2-yl)-2,5-diphenyltetrazolium bromide
MUC20: Mucin 20
NEAA: Non-essential amino acids
NF-κB: Nuclear factor kappa-light-chain-enhancer of activated B cells
OCR: Oxygen consumption rate
OD: Optical density
PEAR: Paired-End reAd mergeR
PITPNM3: Phosphatidylinositol transfer protein 3
P/S: Penicillin/streptomycin
PCA: Principal-component-analysis
PYG: Peptone yeast extract glucose
qPCR: Quantitative real-time polymerase chain reaction
RCM: Reinforced clostridial medium
SCFA: Short-chain fatty acid
SLC2A5: Solute carrier family 2 member 5
SD: standard deviation
TGF-*β*: Transforming growth factor-*β*
TIM-3: T-cell immunoglobulin and mucin domain-3
TPM: Transcripts per kilobase million
TSP-1: Thrombospondin-1
TSB: Tryptic soy broth
TNFSF9: Tumor necrosis factor superfamily member 9
UHPLC-MS/MS: Ultra-high performance liquid chromatography–tandem mass spectrometry
ZO-1: Zonula occludens-1
