# Supplementary material for: A stratified human colon model reveals SCFA cross-feeding-driven host–microbe dynamics associated with epithelial and immune responses
Source: Gut Microbes. 2026 Oct 23;18(1):2749954. doi: 10.1080/19490976.2026.2749954 (PMC13699875; doi:10.1080/19490976.2026.2749954)
Supplement: Supplementary file 1 [file KGMI_A_2749954_SM2476.docx]

Supplementary Materials for

**A Stratified Human Colon Model Reveals SCFA Cross-Feeding-Driven Host-Microbe Dynamics Associated with Epithelial and Immune Responses**

Taewon Kim and Joon-Young Park *et al*.

Corresponding authors: Bong-Soo Kim, bkim79@ewha.ac.kr; Nam Joo Kang, njkang@catholic.ac.kr

**Supplementary Figures**

**Figure S1.** Medium selection and evaluation of growth and SCFA production by *A. caccae* and *B. bifidum* under monoculture and co-culture conditions.

**Figure S2.** Optimizing inoculum concentration and incubation time for SCFA cross-feeder co-culture.

**Figure S3.** Epithelial barrier integrity and junctional organization in Mm 3D-HaeMan.

**Figure S4.** Optimization of expanded strain co-culture condition in Mm 3D-HaeMan.

**Figure S5.** SCFA concentrations, SCFA-related pathway expression, and differential bacterial gene expression across co-culture conditions.

**Figure S6.** MTT-based assessment of cellular metabolic activity in CCD841CoN cells exposed to live, heat-killed, and supernatant fractions of gut bacteria.

**Description of the Supplementary Tables for this Manuscript**

**(provided as a separate file)**

**Dataset 1-10**

**Dataset 1.** Oxygen concentration in Mm 3D-HaeMan.

**Dataset 2.** Coculture optimization of host cells and bacteria.

**Dataset 3.** Bacterial abundance and metatranscriptomic analysis.

**Dataset 4.** Host phenotypic changes after SCFA cross-feeder-coculture.

**Dataset 5.** Host transcriptomic analysis.

**Dataset 6.** Optimization of coculture media and time-course measurements of *AcBb* growth and SCFA production.

**Dataset 7.** Epithelial barrier integrity and junctional organization.

**Dataset 8.** Expanded strain panel dataset for Mm 3D-HaeMan.

**Dataset 9.** Bacterial metatranscriptome changes in presence or absence of host cells.

**Dataset 10.** MTT-based assessment of cellular metabolic activity.

**Supplementary Figures**

**Figure S1. Medium selection and evaluation of growth and SCFA production by *A. caccae* and *B. bifidum* under monoculture and co-culture conditions.** **(a and b)** Growth of *A. caccae* and *B. bifidum* under monoculture and co-culture conditions in four media (RCM, PYG, MRS, and TSB), assessed by OD_600_ and species-specific qPCR (CFU/mL). OD_600_ and qPCR were interpreted as complementary rather than interchangeable measurement, as OD_600_ reflects total turbidity or particle-associated biomass, whereas species-specific qPCR estimates DNA-based abundance of each bacterial species. Statistical comparisons were performed using Dunn’s test with false discovery rate correction. **(c)** Acetate and butyrate concentrations normalized to bacterial cell counts under *AcBb* co-culture conditions in MRS and TSB media. Bars represent the mean, and error bars indicate the standard deviation. Statistical significance was evaluated using a *t*-test with Bonferroni correction. (d) Bacterial amounts were determined at 0, 12, 24 h of cultivation and expressed as log_10_(CFU/mL). Lines indicate temporal changes in *B. bifidum* monoculture, *A. caccae* monoculture, and each strain within the *AcBb* co-culture. Points represent the mean log_10_(CFU/mL), and error bars indicate the standard deviation. Statistical comparison among culture conditions were performed using Dunn’s test. (e) Acetate and butyrate concentrations were measured at 12 and 24 h in *B. bifidum* monoculture, *A. caccae* monoculture, and *AcBb* co-culture conditions (n = 4/group). Bars represent the mean concentration, and error bars indicate the standard deviation. Statistical comparisons among culture conditions were performed using Dunn’s test. *, *p* < 0.05; **, *p* < 0.01; ***, *p* < 0.001.

**Figure S2. Optimizing inoculum concentration and incubation time for SCFA cross-feeder co-culture.** The inoculum levels of *Anaerostipes caccae* and *Bifidobacterium bifidum* for co-culture with host cells were optimized using a small-scale TSB-Gelrite co-culture system. **(a)** Growth of *A. caccae* and **(b)** *B. bifidum* at varying inoculation densities over time in TSB-Gelrite medium. **(c)** Time- and concentration-dependent evaluation of *AcBb* co-cultured in TSB-Gelrite medium, used to identify optimal conditions for application to the Mm 3D-HaeMan system. Optimal inoculum levels were defined based on the following criteria: (1) structural integrity of the bacterial compartment maintained without complete layer collapse, even if minor surface cracking or bubble formation associated with CO₂ production was present; and (2) macroscopically visible and distinguishable bacterial colony formation within the semi-solid layer. Conditions in which extensive matrix fracturing caused loss of compartment boundary definition, or in which bacterial growth was insufficient for visual identification, were classified as non-viable.

**Figure S3. Epithelial barrier integrity and junctional organization in Mm 3D-HaeMan. (a)** Epithelial barrier integrity of CCD841CoN cells under conventional and Mm 3D-HaeMan co-culture conditions. Time-dependent TEER (left panel) and V*te* (right panel) measurements at 0 h (prior to co-culture initiation) and 18 h (end of co-culture period) across experimental groups. Data represent mean ± SD (n = 2–4). *, *p* < 0.05 vs. CC using Welch’s *t*-test. **(b)** Immunofluorescence staining for ZO-1 (red) and nuclei (DAPI, blue) in CCD841CoN cells cultured under CC, Cont, and *AcBb* co-culture conditions. Left panels show XY-plane images; right panels show Z-stack reconstructions along the XZ-axis. Scale bar: 50 μm. CC, conventional culture; TEER, transepithelial electrical resistance; V*te*, transepithelial voltage.

**Figure S4. Optimization of expanded strain co-culture condition in Mm 3D-HaeMan. (a)** Representative image of the Mm 3D-HaeMan system at 0 h and 18 h following inoculation with increasing densities of *F. prausnitzii* and *B. bifidum* (1×10^4^, 2.5×10^4^, and 5×10^4^ cell/bottle for *Fp*; 1×10^2^, 2.5×10^2^, 5×10^2^ cell/bottle for *Bb*). **(b)** Viability and density of CCD841CoN cells (upper panels) and hPBMCs (lower panels) following 18 h of co-culture with *FpBa* at the indicated inoculum levels relative to total cell number. Data represent mean ± SD (n = 3). *, *p* < 0.05 vs. Cont using Welch’s *t*-test. (c) Bacterial cell counts (CFU/mL) of *F. prausnitzii* and *B. adolescentis* collected from the bacterial compartment following 18 h of co-culture under each host cell combination: *FpBa* alone, *FpBa* with CCD841CoN, *FpBa* with hPBMCs, and *FpBa* with both cell types. Data represent mean ± SD (n = 2**–**3). Statistical comparisons were performed using Dunn’s test with false discovery rate correction. *Fp*, *Faecalibacterium prausnitzii*; *Ba*, *Bifidobacterium adolescentis*; hPBMC, human peripheral blood mononuclear cells.

**Figure S5. SCFA concentrations, SCFA-related pathway expression, and differential bacterial gene expression across co-culture conditions.** **(a)** Concentrations of acetate, butyrate, propionate, and succinate in the bacterial compartment across *AcBb*-containing co-culture conditions and *AcBb*-free controls (n = 3/group). **(b)** Pathway-level transcript abundance of butanoate metabolism (ko00650) and propionate metabolism (ko00640) across bacterial culture and host cell co-culture conditions (n = 3/group). **(c)** Volcano plot showing differentially expressed bacterial transcripts across co-culture conditions. Gene families identified by Multivariate association with linear models 2 (MaAsLin2) with |coefficient| > 1 and adjusted *p* < 0.05 (gray dashed line) were considered significantly altered (n = 3/group). Gray dashed lines indicated the significance thresholds.

**Figure S6. MTT-based assessment of cellular metabolic activity in CCD841CoN cells exposed to live, heat-killed, and supernatant fractions of gut bacteria.** CCD841CoN cells were treated with live bacteria (Live), heat-killed bacteria (Dead), or culture supernatants (Sup) prepared from *A. caccae* (Ac), *B. bifidum* (Bb), or the *AcBb* consortium at an inoculum level equivalent to *AcBb*-L, following 18 h cultivation in TSB broth. Culture medium alone (Medium) served as a blank. Relative cellular reductive activity is expressed as a percentage of the untreated control. Data represent mean ± SD (n = 3). *, *p* < 0.05; **, *p* < 0.01 vs. untreated control using Welch’s *t*-test. Ac, *Anaerostipes caccae*; Bb, *Bifidobacterium bifidum*; *AcBb*, *A. caccae* + *B. bifidum* consortium; Sup, culture supernatant; Medium, TSB culture medium.

**Description of the Supplementary Tables for the Manuscript (provided as a separate file)**

**Dataset 1. Oxygen concentration in Mm 3D-HaeMan.** Dissolved oxygen concentration was measured at 1,000 µm intervals from the surface of the host cell culture medium (depth = 0 µm) to the bottom of the semi-solid layer (depth = 25,000 µm) using the Microx 4 oxygen sensor at 0 and 18 h.

**Dataset 2. Coculture optimization of host cells and bacteria.** Viability and density of CCD841CoN cells and human peripheral blood mononuclear cells (hPBMCs) following 18-h co-culture — in TSB, MRS-based solid media; in Mm 3D-HaeMan; with low and high *AcBb* inocula. Bacterial abundance in the presence or absence of host cells.

**Dataset 3. Bacterial abundance and metatranscriptomic analysis.** Quantification of *AcBb* abundance in the semi-solid layer under four co-culture conditions, assessed using species-specific qPCR; Visualized data for Venn diagram showing shared and condition-specific bacterial transcripts; Visualized data for heatmap of significantly altered bacterial transcripts.

**Dataset 4. Host phenotypic changes after SCFA cross-feeder-coculture.** Mitochondrial respiration of CCD841CoN cells was analyzed using Seahorse XFp Mito Stress Test; Expression of tight-junction proteins in CCD841CoN cells co-cultured with *AcBb*; Cytokine profiles of conditioned medium from CCD841CoN cells and hPBMCs co-cultured with *AcBb*; Quantitative cytokine profiling by cytometric bead array.

**Dataset 5. Host transcriptomic analysis.** Visualized data for principal-component-analysis (PCA) of transcriptomes from CCD841CoN cells co-cultured with *AcBb*; Visualized data for volcano plot showing differential expression between *AcBb*-co-cultured and control CCD841CoN cells; Expression levels of DEGs associated with cellular energy metabolism, intestinal barrier function, and cell signaling pathways; GO enrichment analysis of DEGs from *AcBb* co-cultured CCD841CoN cells.

**Dataset 6. Optimization of coculture media** **and time-course measurements of *AcBb* growth and SCFA production.** Growth of *A. caccae* and *B. bifidum* under monoculture and co-culture conditions in four media (RCM, PYG, MRS, and TSB); Produced SCFA concentrations under co-culture conditions in MRS and TSB media; Time-course measurements of bacterial abundance and acetate/butyrate concentrations in *A. caccae* monoculture, *B. bifidum* monoculture, and *AcBb* co-culture in TSB medium.

**Dataset 7. Epithelial barrier integrity and junctional organization.** Epithelial barrier integrity of CCD841CoN cells under conventional culture and Mm 3D-HaeMan co-culture conditions. Time-dependent TEER and V*te* measurements at 0 h and 18 h across experimental groups.

**Dataset 8. Expanded strain panel dataset for Mm 3D-HaeMan.** Host cell viability and density after co-culture with *F. prausnitzii* and *B. adolescentis* in Mm 3D-HaeMan. Bacterial cell counts (CFU/mL) of *F. prausnitzii* and *B. adolescentis* collected from the bacterial compartment following 18 h of co-culture under each host cell combination

**Dataset 9. Bacterial metatranscriptome changes in presence or absence of host cells.** Visualized data for volcano plot showing differentially expressed bacterial transcripts across co-culture conditions in the presence or absence of CCD841CoN or hPBMCs.

**Dataset 10. MTT-based assessment of cellular metabolic activity.** CCD841CoN cells were treated with live, heat-killed bacteria, or culture supernatants prepared from *A. caccae*, *B. bifidum*, or the *AcBb* consortium at an inoculum level equivalent to *AcBb*-L, following 18 h cultivation in TSB broth. Culture medium alone (Medium) served as a blank. Relative cellular reductive activity is expressed as a percentage of the untreated control.
